# Mouse olfactory system acts as anemo-detector and anemo-discriminator

**DOI:** 10.1126/sciadv.adq8390

**Published:** 2025-10-08

**Authors:** Sarang Mahajan, Susobhan Das, Suhel Tamboli, Sanyukta Pandey, Anindya S. Bhattacharjee, Meenakshi Pardasani, Priyadharshini Srikanth, Shruti D. Marathe, Avi Adlakha, Lavanya Ranjan, Nixon M. Abraham

**Affiliations:** Laboratory of Neural Circuits and Behaviour (LNCB), Department of Biology, Indian Institute of Science Education and Research (IISER), Pune, Maharashtra, 411008, India.

## Abstract

Airflow detection while smelling is a fundamental requirement for olfaction, yet the mechanisms underlying such multimodal processing in the olfactory system remain unknown. We report here that mice can accurately discriminate airflow in the absence of whiskers. Modulated sniffing and refined calcium signaling in the olfactory bulb inhibitory network during olfactory anemo-discriminations confirmed the orthonasal airflow information processing. Genetic perturbation of AMPAR function and optogenetic control bidirectionally shifted the anemo-discrimination learning pace, with contrasting phenotypes observed for odor learning, engagement of inhibitory circuits, and setting the optimal inhibition level for stimulus refinement. Enhanced learning caused by multimodal odor-airflow stimuli at subthreshold levels confirmed the heightened olfactory perception by mechanical stimuli. Our results thus explain the multimodality of olfaction and reveal the unexplored dimensionality of odor perception.

## Teaser

The mouse olfactory system can sense, refine, and process information pertaining to both mechanical and chemical cues.

## INTRODUCTION

The multisensory environment renders the brain capable of perceiving the external world by combining concurrently occurring diverse sensory stimuli. While humans rely vastly on visual and auditory inputs, rodents primarily use olfactory and vibrissal cues from their surroundings. In nature, odor plumes carry odorant molecules associated with varying airflows. What is the role of carrier airflow in olfactory perception? The hypothesis of mechanosensation through the olfactory system emerged from the seminal works of Adrian (*1*) and Ueki and Domino (*2*). Evidence from olfactory bulb (OB) recordings, which detected neural activity even in the absence of odorant stimuli, further supported this notion (*3*, *4*). Recently, this hypothesis was tested by recording electrical activity from olfactory sensory neurons (OSNs) in response to mechanical stimuli (*5*, *6*). OSNs in the rodent nose detect and transfer both mechanical (*5*, *6*) and odorant information to the OB (*7*, *8*). In the OB, signaling through mitral and/or tufted cells is refined by the inhibitory network (*9*, *10*) and top-down modulatory inputs (*11*). Hence, the OB acts as an early site for integrating odorant- and airflow-related information in mammals. Despite the existing literature on the cellular mechanisms of inhibitory interactions (*9*, *10*, *12*), their functional relevance in processing combined olfactory and mechanosensory information remains largely unknown.

Sniffing is integral to olfactory perception, not only for gathering volatile information from the surroundings but also for conveying socially relevant cues, such as hierarchical status in animals (*13*–*17*). Respiration-coupled activity in various olfactory pathway centers has been reported since Adrian’s seminal experiments (*18*). While odor representation in the OB (*19*–*22*) and the piriform cortex (*23*) can be sniffing-dependent, what information could be coded by the modulations of respiration in the absence of odorants? Olfactory perception is tightly coupled to respiratory patterns (*20*). The rhythmic activity of the neurons in the pre-Bötzinger complex may regulate the mechanical drive needed for active sampling of odorants (*24*, *25*). The oscillatory activity in the OB projection neurons, generated by the sniffing-induced mechanosensation in a glomerulus-specific manner, is crucial for phase coding of odor identity (*26*). Despite these findings, the neural mechanisms underlying mechanosensation in OB circuits remain undiscovered.

How do OB circuits maintain a robust phase code? What is the role of OB inhibitory circuits in regulating mechanosensation in the olfactory system? Airflow elicits broader activity patterns in the OB compared to odor-evoked responses (*27*), implying greater recruitment of inhibitory networks for airflow signals. A specific odorant molecule activates a subset of OSNs, resulting in distinct odor-evoked glomerular activity patterns. OB inhibitory circuits modulate the spiking activity of projection neurons and help refine odorant information conveyed to higher centers (*9*, *12*). As airflow evokes broader activation, the optimized inhibition needed to refine the information differs from those for odor signals. Hence, probing the role of the inhibitory network is pivotal for understanding the fundamental principles of mechanosensation through the rodent olfactory system.

Here, we show that mice can detect and discriminate ethologically relevant airflow rates through their nose. In vivo Ca^2+^ imaging from inhibitory interneurons, combined with genetic manipulations [e.g., deletion of the GluA2 subunit of AMPA receptors (AMPARs)] and optogenetic approaches [channelrhodopsin-2 (ChR2) and archeorhodopsin (Arch) activations], confirmed the role of OB circuits in processing and refining airflow information. We also elucidated that the optimal inhibition needed for refining odor versus airflow discrimination differs substantially, indicating the varying recruitment of inhibitory circuits by chemical and mechanical stimuli. In addition, we show that airflow-related mechanosensory inputs enhance olfactory perception at subthreshold levels. Together, these results prove that the mouse olfactory system can act as an anemo-detector and anemo-discriminator.

## RESULTS

### Mice can detect and discriminate diverse airflow rates using their nose, even in the absence of whiskers

**Fig. 1.**
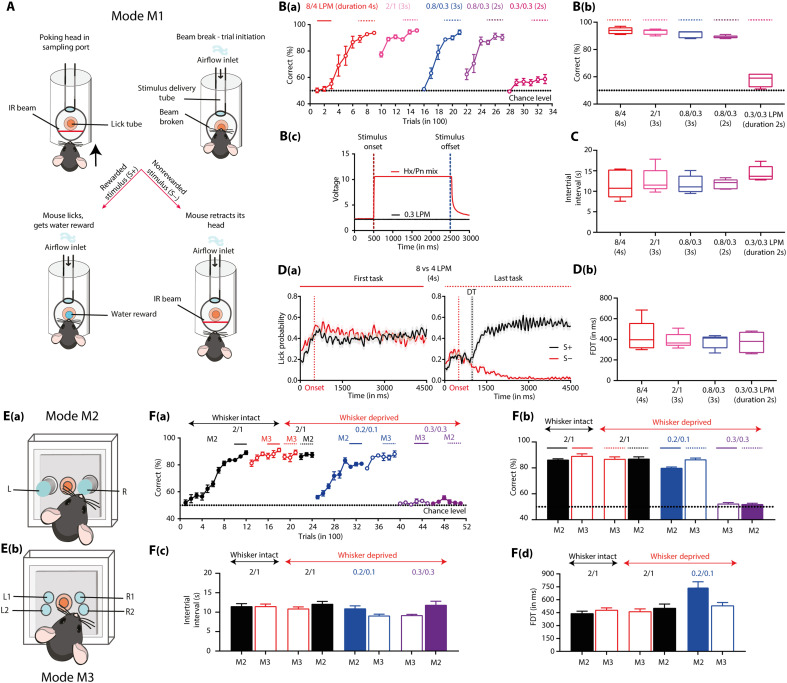
Mice can detect and discriminate airflows in the absence of whiskers. (**A**) Go/no-go operant conditioning paradigm (M1 mode). Trials begin when a mouse pokes its head into the sampling port, activating an IR beam. The animal samples the stimulus. For rewarded stimuli (S+), licking triggers a water reward. For unrewarded stimuli (S−), trained animals withdraw without licking. (**B**) Animals can detect and discriminate different airflow pairs in the 2-s stimulus duration. [B(a)] Airflow discrimination learning accuracies measured for diverse airflow pairs at different stimulus durations (black dotted line at *y* = 50 is the chance level). [B(b)] Average learning accuracies measured from the last 300 trials for various airflows. The accuracy exceeds 80% for most pairs, except 0.3 LPM versus 0.3 LPM (chance level ~ 50%). [B(c)] The PID profile of airflows and odors reveals the absence of odor stimulus in the airflow. Comparison of PID profiles of airflow (0.3 LPM, black) and odor (hexanal-pentanone mixture, red). (**C**) ITIs measured from the last 300 trials. ITIs were 11 to 14 s, representing optimal motivation. (**D**) FDT for airflow discrimination tasks calculated from lick patterns. [D(a)] Representative lick responses of a single animal averaged over 300 trials (150 S+ and 150 S−) during the first and last tasks (the shaded region represents SEM, and the red dotted vertical line is the stimulus onset). [D(b)] Average FDTs for different airflow pairs were in the range of 300 to 500. (**E**) Different modes of stimulus delivery. (**F**) Animals can discriminate the airflow pairs in the absence of whiskers. [F(a)] Training schedule and airflow discrimination accuracies. [F(b)] Learning accuracies measured from the last 300 trials. [F(c)] ITIs measured from the last 300 trials were in the optimum range of 9 to 12 s. [F(d)] Average FDTs for different airflow pairs under different stimulus delivery and WD conditions were in the range of 300 to 500 ms.

The less-explored dimensions of the boundless chemical world make the olfactory stimulus space challenging to resolve (*28*–*30*). Variations in odor stimuli because of mechanical (airflow-induced) and thermal (temperature-driven changes in volatility) factors further contribute to the uniqueness of olfactory perception. To investigate how mechanosensation modulates olfactory perception, we started by studying mice’s discrimination behavior in the absence of any odor molecule. To achieve this, we built a customized instrument capable of providing airflows as stimuli in three different modes (M1, M2, and M3; Fig. 1). Given that airflow stimuli can activate the whisker system in mice, these stimulus delivery modes were designed to allow whisker activation. We started with training the animals to discriminate the airflows using M1 mode, 8 liters per minute (LPM) versus 4 LPM [airflow (4 s) is delivered from the top; Fig. 1A]. These flow rates were strong enough to cause deflection of a plucked whisker. Animals reached the asymptotic levels of learning (accuracy >85%) within 900 trials [Fig. 1B(a)]. Having observed their discrimination abilities, we continued the training on various airflow pairs to optimize the stimulus strength and duration. Mice successfully learned to discriminate different airflow pairs within 600 to 900 trials, reaching ~90% accuracy toward the end of training [2 LPM versus 1 LPM, 3-s duration; 0.8 LPM versus 0.3 LPM, 3-s duration; 0.8 LPM versus 0.3 LPM, 2-s duration; Fig. 1B, (a) and (b)]. To exclude the possibility of using any nonspecific cues while discriminating airflows, mice were trained on a control task (0.3 LPM versus 0.3 LPM, 2-s duration), wherein their performance was at the chance level [Fig. 1B, (a) and (b)]. This was further confirmed by the photoionization detector (PID) analysis of various airflows and odors. We did not observe any notable voltage shifts for airflows, whereas a well-defined voltage shift was observed during the odor stimulus [Fig. 1B(c) and fig. S1]. Animals were optimally motivated to perform these tasks, as quantified by intertrial intervals (ITIs) (Fig. 1C). As mice performed discrimination of different flow pairs with high accuracy, we quantified another behavioral readout, i.e., time taken by mice to discriminate various flow pairs. This was calculated from the lick patterns shown by animals toward rewarded and nonrewarded airflow stimuli [Fig. 1D(a)]. Our results show comparable flow discrimination time (FDT) across different airflow pairs, making the sensing mechanism and the underlying neural pathways an interesting open question [Fig. 1D(b), one-way repeated-measures analysis of variance (ANOVA), *F* = 0.4158, *P* = 0.6725].

Having observed mice’s airflow discrimination abilities, we tried to deliver the airflow stimulus dissociated from the reward delivery tube to monitor their sampling strategies with more precision. We delivered the flows from two different focal points in one mode [M2; Fig. 1E(a)] and in a more diffused manner in the second mode [M3; Fig. 1E(b)] and trained the animals to discriminate 2 LPM versus 1 LPM. Mice reached the asymptotic phase of learning within 1200 trials, with final accuracies similar across M2 and M3 modes [Fig. 1F, (a) and (b): M2 mode: 86.33 ± 0.7182; M3 mode: 89.21 ± 1.650; two-tailed paired *t* test, *P* = 0.1348]. From our video analysis, we observed mice using their nose to sample the airflows during the airflow discrimination task (movies S1 and S2 and fig. S2).

To test whether airflow discrimination relied on whiskers, we trimmed them and retrained the mice to discriminate 2 LPM versus 1 LPM airflow. Mice were able to discriminate these airflows with high accuracy in the absence of whiskers [Fig. 1F, (a) and (b): M2 mode: whiskers-intact (WI), 86.33 ± 0.7182; whiskers-deprived (WD), 87.14 ± 1.438; two-tailed paired *t* test, *P* = 0.6135; M3 mode: WI, 89.21 ± 1.650; WD, 86.90 ± 1.628; two-tailed paired *t* test, *P* = 0.3024]. Similar results were observed when mice discriminated lower-strength airflows (0.2 LPM versus 0.1 LPM). Mice failed to discriminate when they were challenged with the same airflow rates [0.3 LPM versus 0.3 LPM; Fig. 1F, (a) and (b)]. The efficacy of animals discriminating airflow rates in the absence of whiskers was also demonstrated by another method of whisker deprivation, i.e., whisker plucking, wherein the performance of animals was not different before and after the deprivation (fig. S3). These results confirm mice’s ability to discriminate various airflow rates in the absence of whiskers and using their nose [Fig. 1F(a)]. High-accuracy performances were observed in WI and WD mice at the end of each training session, except the chance level performance observed for the control task that involved the discrimination of same airflow rates [Fig. 1F(b)]. Furthermore, to rule out the possibility of animals using nonspecific residual odor cues for discrimination, we trained them to distinguish airflows delivered from a zero-air cylinder. The animals performed with >80% accuracy in this task (fig. S4A). In addition, we conducted airflow discrimination tasks under head-restrained conditions, directing the stimulus either to the nostrils or to the caudal region, away from the nostrils. While the animals learned to discriminate the stimuli directed to the nostrils, mice were unable to learn the stimuli directed to the caudal region (fig. S4B), thereby ruling out the possibility of using nonspecific cues in airflow discrimination. Mice showed optimal motivation levels, quantified by ITIs, under WI and WD conditions [Fig. 1F(c)]. We further analyzed the FDTs from various airflow discriminations and found similar FDTs in WI and WD mice, ruling out the possible involvement of the whisker system in the observed anemo-discrimination behavior [Fig. 1F(d), M2 and M3 mode, before and after whisker deprivation, two-tailed paired *t* test, *P* = 0.3589 and *P* = 0.6627, respectively].

### Olfactory information processing centers are involved in airflow rate discrimination

**Fig. 2.**
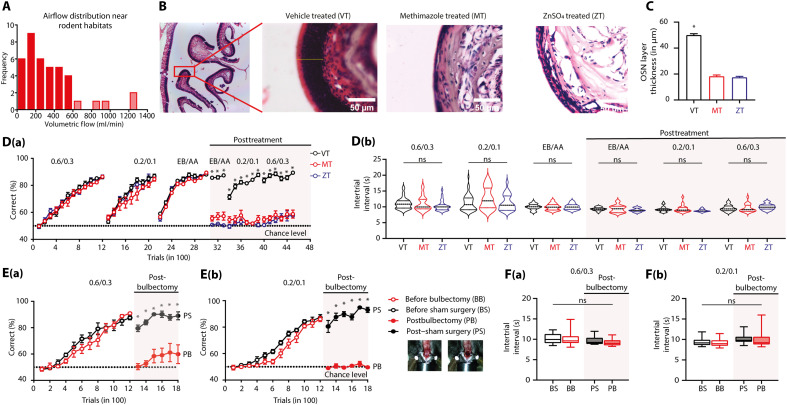
Mice use the olfactory system to detect and discriminate the airflows. (**A**) Histogram representing the distribution of airflows in potential rodent habitats measured using hot-wire anemometer from 40 different locations. A total of 90% of airflow rates was in between 0.1 and 0.6 LPM (shown by solid red bars), with 0.35 LPM being the average airflow rate. (**B**) Images showing hematoxylin and eosin–stained sections of the olfactory epithelium under different treatment conditions. (**C**) Thickness of epithelium measured across vehicle-, methimazole-, and ZnSO_4_-administered animals after 2 days of the treatment. The epithelium thickness for vehicle (VT, vehicle treated) was significantly higher as compared to methimazole-treated (MT) and ZnSO_4_-treated (ZT) animals (one-way ANOVA, *F* = 354.2, *P* < 0.0001, *n* = 2 mice for all groups, *n* = 50 ROIs from six sections from each mouse). (**D**) [D(a)] Learning accuracies of animals for different airflow and odor pairs before and after the treatment. The accuracies of animals that underwent ZnSO_4_ and methimazole treatments were significantly lower than those of the control group. [D(b)] Violin plots representing the ITI of animals. The ITI of all animals throughout the training remained in the range of 9 to 11 s. A *P* value of >0.05 was observed across different groups for different airflow and odor pairs. ns, not significant. (**E**) [E(a)] Learning accuracies of animals for 0.6 LPM versus 0.3 LPM and [E(b)] 0.2 LPM versus 0.1 LPM before and after the bulbectomy surgery. Before surgery, both bulbectomized and sham surgery groups showed similar learning. Following surgery, the performance of the bulbectomized group dropped to the chance level. However, the sham surgery group did not show any performance deficit (*P < 0.05). (**F**) [F(a)] ITIs of different groups of animals for 0.6 LPM versus 0.3 LPM and [F(b)] for 0.2 LPM versus 0.1 LPM before and after the surgery. The ITI of the animals did not reveal any significant differences across.

As we demonstrated the involvement of the rodent nose in discriminating a large range of airflow rates, we next aimed to delineate the role of olfactory centers in processing ethologically relevant airflow rate information. As a first step, we quantified airflow distribution in rodent habitats across different locations using a hot-wire anemometer. Measurements from 40 locations revealed that 90% of measured airflow lays in the range of 0.1 to 0.6 LPM (Fig. 2A). This airflow range was mostly considered further for designing the stimulus pairs in anemo-discrimination experiments.

The role of the olfactory epithelium in airflow rate discrimination was investigated by ablating OSNs using zinc sulfate (ZnSO_4_) intranasal and methimazole (an olfactotoxic drug) intraperitoneal injections, both known to induce hyposmia/anosmia in animals (*31*, *32*). These methods have been used earlier to ablate OSNs, where the reduction in OSN layer thickness was used to validate the effect (*33*, *34*). A significant reduction in the thickness of the OSN layer was observed 2 days after the treatments, indicating the efficiency of these methods in ablating OSNs (Fig. 2, B and C; vehicle, 49.95 ± 1.110; methimazole, 18.24 ± 1.035; ZnSO_4_, 17.41 ± 0.7997; one-way ANOVA, *F* = 354.2, *P* < 0.0001). To assess the involvement of OSNs in anemo-discrimination, three batches of mice were trained on two airflow discrimination tasks (0.2 LPM versus 0.1 LPM and 0.6 LPM versus 0.3 LPM) and an odor discrimination task (amyl acetate versus ethyl butyrate using the same flow rate, 0.6 LPM). All groups of mice learned to discriminate the airflow rates and odors with similar accuracy [Fig. 2D(a), two-way ANOVA with Tukey’s multiple comparison, *P* > 0.05]. These mice were then administered with the following: 5% ZnSO_4_ (intranasal application, group 1), methimazole (intraperitoneal injection, group 2), and phosphate-buffered saline (PBS; intranasal application and intraperitoneal injections, group 3). The performance of animals was then tested on previously learned odor and airflow rate discrimination tasks. While the performance of treated mice dropped to the chance level, control mice performed with an accuracy of >80% in a previously learned odor discrimination task, thereby confirming the ablation of OSNs. Upon repeating airflow discrimination training, the control group performed with high accuracy, while the treated animals performed at chance levels [Fig. 2D(a), two-way ANOVA with Tukey’s multiple comparison, *P < 0.05]. The ITIs, a measure of animals’ motivation, remained optimal and unaltered even after the treatments [Fig. 2D(b), one-way ANOVA for individual airflow/odor pairs, *P* > 0.05]. These results imply the potential role of the main olfactory epithelium in anemo-discrimination.

Once we confirmed the olfactory route of airflow sensation, we investigated the role of OB, the first relay station in olfactory signaling, in processing airflow rate-related information. Mice’s airflow discrimination abilities were measured before and after surgically removing the OB. Two groups of experimental mice and corresponding control animals (sham surgery) were trained to discriminate 0.2 LPM versus 0.1 LPM and 0.6 LPM versus 0.3 LPM. Animals performed with >80% accuracy within 1200 trials for both airflow pairs. Experimental and control mice did not show any difference in learning pace before the treatment [Fig. 2E, (a) and (b), two-way ANOVA with Tukey’s multiple comparison, *P* > 0.05 for both groups]. Once the animals reached the asymptotic phase of learning, the OB was surgically aspirated, and animals were allowed a recovery period of 12 to 15 days. Training was then resumed for all mice to discriminate the already learned airflow pair. Postbulbectomy, the performance of experimental mice dropped to chance levels for both airflow pairs, where no alteration in the performance was observed for the control animals that underwent sham surgery [Fig. 2E, (a) and (b), two-way ANOVA with Tukey’s multiple comparison, *P < 0.05]. We observed no differences in the ITIs for all groups of animals for both airflow pairs, indicating that the motivational levels of animals were optimal and comparable before and after the treatment [Fig. 2F, (a) and (b), one-way ANOVA, *P* > 0.05]. These findings confirm the involvement of OB in anemo-discriminations.

### Learning-dependent refinement of sniffing during the decision-making period in airflow rate discrimination

**Fig. 3.**
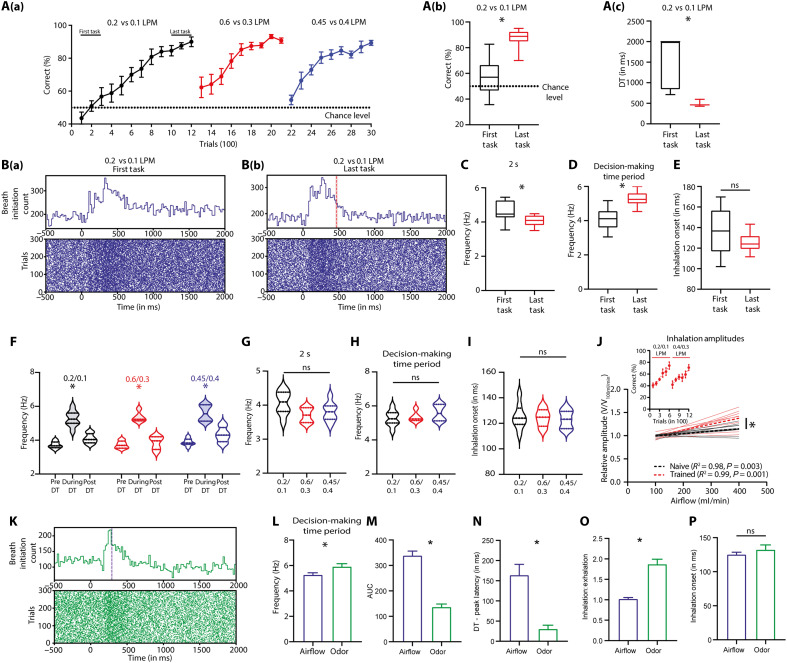
Mice exhibit learning-dependent sniffing refinement during anemo-discrimination. (**A**) [A(a)] Learning curves of animals trained on different airflow pairs (0.2 LPM versus 0.1 LPM, 0.6 LPM versus 0.3 LPM, and 0.45 LPM versus 0.4 LPM). Box-and-whisker plots showing [A(b)] accuracies and [A(c)] FDTs of animals during the first and last tasks of the training for 0.2 LPM versus 0.1 LPM. (**B**) {[B(a)] and [B(b)]} Raster plots and histogram representing the inhalation onset during the first and last tasks for 0.2 LPM versus 0.1 LPM. The red vertical dotted line indicates the average FDT, and the shaded area represents SEM. Box-and-whisker plots showing (**C**) the SF of animals during the 2-s stimulus duration, (**D**) SF of animals during the decision-making window, and (**E**) onset of first inhalation during the decision-making window for the first and last tasks of the training for 0.2 LPM versus 0.1 LPM. Violin plots showing (**F**) SFs of animals before, during, and after the decision-making period, (**G**) SFs of animals during the 2-s stimulus period, (**H**) SFs of animals during the decision-making period, and (**I**) first inhalation onset during the decision-making window across different airflow pairs (*P < 0.05). (**J**) Graph demonstrating an increase in relative amplitudes for inhalation along with stimulus strength in learned (red) and naïve mice (black). (**K**) Raster plots and histograms representing the inhalation onset for odor-based discrimination {binary mixture of enantiomers [octanol (+) versus octanol (−)]} pooled across animals in the last phase of learning (last 300 trials). Bar graphs representing (**L**) SFs, (**M**) AUC, (**N**) difference in the FDT and peak latency, (**O**) ratio of time spent in inhalation to that of the time spent in exhalation, and (**P**) onset of first inhalation of animals for airflow (0.2 LPM versus 0.1 LPM) and odor trials during the decision-making period (*P < 0.05).

As we observed a loss of airflow rate discrimination abilities on blocking the airflow information processing through the olfactory pathway, we next probed modulation in their sampling behavior during airflow rate discriminations. We trained another batch of mice under head-restrained conditions and monitored their sniffing behavior (see Materials and Methods), an exploratory behavior associated with olfaction, during different phases of learning (*35*). Mice were trained on different airflow rates until they reached the performance accuracy of >80% [0.2 LPM versus 0.1 LPM (1200 trials), 0.6 LPM versus 0.3 LPM (900 trials), and 0.4 LPM versus 0.45 LPM (900 trials); Fig. 3A]. As animals learned to discriminate the first airflow pair with high accuracy reflected by faster FDTs, we observed substantial changes in breathing modulations, quantified by breath initiation counts (BICs) and other sniffing-associated parameters [sniffing frequency (SF)] (Fig. 3, A to E). The raster plots of breath initiations and corresponding histograms showed that at the early stages of learning (first task), BICs remained higher even after the sniffing peak; however, in the learned phase (last task), BICs were comparable to the prestimulus duration [Fig. 3B, (a) and (b)]. This was further reflected in the SF measurements during the entire stimulus duration and the decision-making period, which is the same as discrimination time (DT). While SF during stimulus duration decreased from the first to last task, SFs during the decision-making period showed a significant enhancement with learning (Fig. 3, C and D, two-tailed paired *t* test, *P < 0.05). However, the inhalation onset remained unchanged and was independent of learning phases (Fig. 3E, two-tailed paired *t* test, *P* = 0.1955). Furthermore, we analyzed SFs before, after, and during FDTs when animals were performing with >80% accuracy for all three airflow pairs they were trained on. Mice showed higher SFs during the FDTs compared to before and after the decision-making duration, and this was independent of reward contingencies (Fig. 3F and fig. S5). The SFs during the entire stimulus duration, SFs during the decision-making period, and inhalation onsets remained similar across all airflow pairs tested (Fig. 3, G to I, one-way repeated-measures ANOVA, *P* > 0.05). In addition, to explore how ambient airflows influence mechanosensation in the olfactory epithelium, we measured the impact of varying airflow strengths on pressure within the nasal cavity of naïve and learned mice. We observed that relative inhalation amplitudes increased with stronger airflows, while exhalation amplitudes showed a weak trend, and SFs remained unchanged (Fig. 3J and fig. S6). These results show how stimulus strength can influence mechanosensation through orthonasal respiration. Our results provide conclusive evidence for the learning-dependent temporal refinement of sniffing confined to the decision-making period, which may help animals in making accurate decisions.

### Distinct sampling behaviors are shown by mice for airflow and odor discrimination

Rodents are known to modulate their sniffing behavior in odor discrimination contexts (*16*, *35*). Given the noticeable sniffing modulations observed in mice during airflow discriminations, we further compared their sampling behaviors during odor and airflow discriminations. We trained a new group of animals on an odor discrimination task where they had to distinguish between a binary mixture of enantiomers [octanol (+) versus octanol (−)]. After the animals reached a performance accuracy of >80%, we quantified various sniffing parameters and compared them with those during airflow discriminations. Mice showed distinct temporal patterns of breathing during airflow and odor discrimination behavior [Fig. 3, B(b) and K]. The SF of animals increased for both airflows and odors during the decision-making window, but the SFs for odors were significantly greater than that for airflows (Fig. 3L, two-tailed unpaired *t* test, *P* = 0.0291). Furthermore, breath initiation histograms revealed that the animals displayed enhanced sniffing for longer durations in the case of airflow discrimination compared to odor discrimination. To further quantify the temporal aspects of breathing, we compared the area under the curve (AUC) during the decision-making period for airflow and odor discriminations. While inhalation and exhalation dynamics were used to calculate SFs, BICs, plotted as a histogram of inhalation onsets over time at a population level, were used to calculate the AUCs. The AUCs for airflows were significantly higher than those of odors (Fig. 3M, two-tailed unpaired *t* test, *P* < 0.0001).

As we observed different DTs for odor and airflow discriminations, we calculated the difference between sniffing peak latency and DT. The difference was greater for airflow discriminations compared to odor discriminations, implying that animals need more time to make airflow rate–based decisions at optimal SFs than odorants (Fig. 3N, two-tailed unpaired *t* test, *P* = 0.0012). Furthermore, to understand breathing dynamics in detail, we analyzed the inhalation-exhalation times shown by animals. During odor discrimination, we observed a significantly higher inhalation-exhalation time ratio than airflow discrimination (Fig. 3O, two-tailed unpaired *t* test, *P* < 0.0001). This may reflect animals’ attempt to collect maximum odor information in a discrimination context. Despite the changes in several sniff parameters, the time of first inhalation remained similar for both the odor and airflow discriminations (Fig. 3P, two-tailed unpaired *t* test, *P* = 0.3419), suggesting that first inhalation happens independently of stimulus characteristics and the temporal dynamics of breathing evolves depending on the type of stimulus. Together, these results indicate the involvement of the mouse olfactory system in anemo-discrimination and prove that animals use distinct sniffing strategies while challenged with airflow rate and odor discriminations.

### The OB inhibitory network regulates anemo-detection and anemo-discrimination

**Fig. 4.**
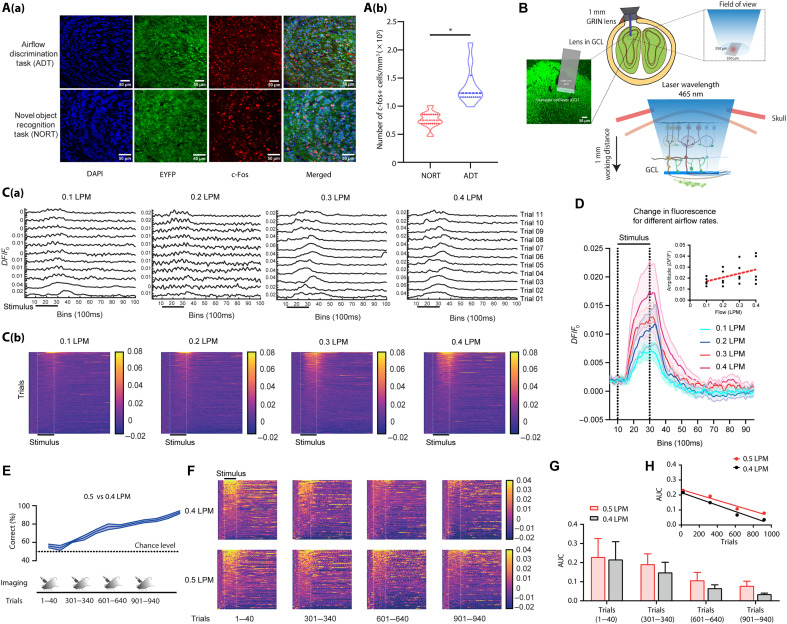
GABAergic (GAD65 + ve) OB interneurons are involved in processing airflow stimuli in a learning-dependent manner. (**A**) [A(a)] c-Fos expression in the OB of animals trained on the airflow discrimination task and NORT. Blue, DAPI staining; green, GAD65-EYFP expression; red, c-Fos expression. [A(b)] Comparison of c-Fos activity in the GCL of animals trained to discriminate airflows showing higher c-Fos activity compared to controls (NORT). (**B**) Illustration representing the scheme of microendoscopic calcium imaging, depth range, and field of view of imaging cannula used for GC imaging. (**C**) [C(a)] Trial-by-trial traces of GAD65 + ve OB interneuron response to different airflows in a single anesthetized animal. [C(b)] Pseudocolor heatmaps of Ca^2+^ dynamics elicited by various airflows. (**D**) Average *DF*/*F*_0_ responses of animals for various airflows. The solid line represents the means, while the shaded area represents SEM. The subgraph depicts the change in amplitude with stimulus strength. The amplitude showed a positive correlation with stimulus strength (Pearson correlation: *R*^2^ = 0.9911, *P* = 0.0045). (**E**) Accuracy of animals with the progression of training. The schematic for the sequence of imaging sessions of the experiment is provided. The imaging is done at the beginning of each task for 40 trials. (**F**) Pseudocolor heatmaps of Ca^2+^ responses elicited by 0.4 and 0.5 LPM for different tasks. The qualitative analysis shows a potential refinement in the Ca^2+^ transients with learning. Data are sorted in decreasing order of Ca^2+^ response during stimulus. (**G**) Bar graphs representing the average AUC for 0.4 and 0.5 LPM with trials. With learning, the AUC for both airflows decreased. (**H**) Plot representing a change in the AUC of animals for 0.4 and 0.5 LPM. For both airflows, the change in AUC showed an inverse correlation with learning (0.4 LPM: *R*^2^ = 0.9726, *P* = 0.0138; 0.5 LPM: *R*^2^ = 0.9636, *P* = 0.0184).

As the surgical removal of the OB led to the inability of mice to execute anemo-processing tasks, we then dissected the underlying circuitry in OB-intact mice. To achieve this, the neuronal activation pattern in the OB following the airflow discrimination task was examined using c-Fos. Neurons in different layers of OB expressed c-Fos, whereas the signal was most abundant in the granule cell layer (GCL) that harbors most of the GAD65 (GAD2)–positive [an isoform of GABA (γ-aminobutyric acid)–synthesizing enzyme] GABAergic inhibitory interneurons. When c-Fos activity in animals trained to discriminate airflows was compared to that of control animals [mice trained on a novel object recognition task (NORT)], higher activity was observed in the GCL of animals in the airflow discrimination group [enhanced yellow fluorescent protein (EYFP) labeled; Fig. 4A, (a) and (b), two-tailed paired *t* test, *P* < 0.0001]. To further explore how the OB inhibitory network responds to airflow stimuli, we quantified Ca^2+^ dynamics using the specific expression of GCaMP6f in GAD65 interneurons. Gradient-index (GRIN) lenses were implanted in the GCL of GAD65-GCaMP6f transgenic mice, and calcium dynamics were monitored while they were presented with various airflow rates (see Materials and Methods and Fig. 4B). The population activity of OB GAD65 interneurons was investigated under both anesthetized and awake conditions. While stimulus strength–dependent activation was studied under anesthesia, the effect of airflow discrimination learning on the interneuronal population activity was monitored during different phases of learning. Upon quantifying fluorescence changes in response to different airflows under anesthetized conditions, the *DF*/*F*_0_ increased after the stimulus onset, reached a peak, and fell back to the baseline [Fig. 4C(a)]. The fluorescence changes were quantified for 250 trials across five animals for various airflow rates [Fig. 4C(b)]. Averaging *DF*/*F*_0_ across all animals revealed that the increase in amplitude correlated with stimulus strength (Fig. 4D; Pearson correlation: *R*^2^ = 0.9911, *P* = 0.0045). This confirms the involvement of OB GABAergic interneurons in processing airflow-related information.

Having observed the involvement of inhibitory network in airflow information processing, we further investigated the modulation of inhibition in different learning phases. A group of mice that express GCaMP6f in GAD65 interneurons was trained to discriminate the airflows at 0.5 LPM versus 0.4 LPM (1200 trials, four tasks) under head-restrained conditions. Calcium activity was recorded from the first 40 trials of each task (300 trials) from each animal. Animals learned to discriminate this flow pair with a high accuracy of >80% (Fig. 4E). We observed calcium activity in response to both rewarded and nonrewarded stimuli throughout the discrimination training duration, however, in a decreasing manner as the learning progressed (Fig. 4, E and F). To quantify this Ca^2+^ dynamics, the AUCs from the *DF*/*F*_0_ traces for both rewarded and nonrewarded stimuli were calculated. A decrease in AUCs was observed for both stimuli as the accuracy of discrimination performance improved from the chance level to ~90% correct responses (Fig. 4, G and H; 0.4 LPM: *R*^2^ = 0.9726, *P* = 0.0138; 0.5 LPM: *R*^2^ = 0.9636, *P* = 0.0184). We observed a similar trend in the reduction of AUCs from the “not-learned” state to a “learned” state during a go/no-go complex odor discrimination task as well (fig. S7). This suggests that the interneuron network is actively involved in refining both odor and airflow discrimination tasks. These findings provide the first experimental evidence for the modulation of OB inhibitory interneuronal population activity during the airflow discrimination in a learning-dependent fashion.

### Synaptic inhibition in the mouse OB controls anemo-discrimination behavior

**Fig. 5.**
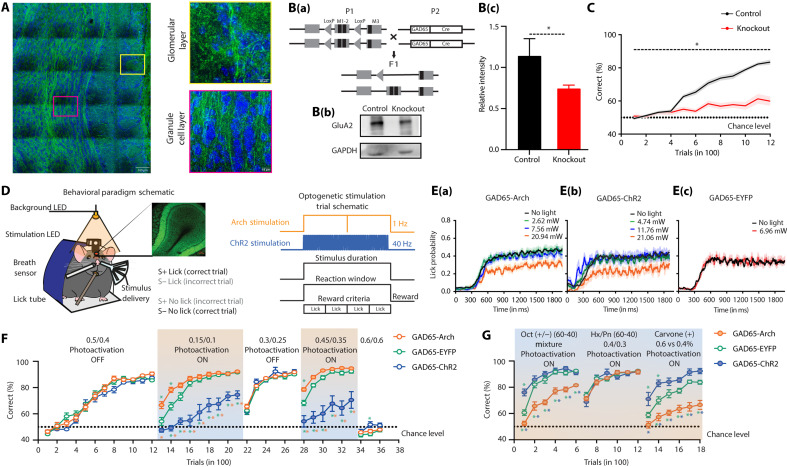
Synaptic inhibition in the mouse OB controls anemo-detection and anemo-discrimination. (**A**) Tile scan representing GAD65 (GAD2) expression in the OB. Enlarged images (toward the right) show expression in different layers of OB. Green, GAD65; blue, DAPI. (**B**) [B(a)] Scheme of genetic crosses to create GAD65-GluA2 knockout animals. [B(b)] Western blot showing GluA2 expression in the OB of control and GAD65-GluA2 knockout animals. The GAPDH protein band is observed in OB lysates of both groups. [B(c)] Relative GluA2 expression in control animals (normalized with corresponding GAPDH). GluA2 expression is reduced significantly in knockout animals. (**C**) Comparison of learning curves for control and GAD65-GluA2 knockout animals trained to discriminate 0.5 LPM versus 0.4 LPM. GAD65-GluA2 knockout animals showed significantly lower learning accuracy. (**D**) (Left) Experimental setup to perform behavioral training under head-restrained conditions. (Right) Illustration representing optogenetic (LED) stimulation. A 595-nm amber LED is fixed on top of a circular cranial window over the OB in a GAD65-Arch–expressing mouse and a 473-nm blue LED in a GAD65-ChR2–expressing mouse. The response window, wherein the animal has to lick to get the reward, coincides with the stimulus delivery period. (**E**) LED power optimization for optogenetic modification of GAD65 + ve interneurons during an airflow detection task. Average lick responses of [E(a)] GAD65-Arch and [E(b)] GAD65-ChR2 mice to different light intensities. Because each mouse licked differentially at different LED powers, the LED power for further discrimination experiments was determined individually. [E(c)] Average lick responses of GAD65-EYFP mice at no light and light at 6.96 mW (average light intensity used for ChR2 animals). The solid lines and shaded areas represent the means and SEM. (**F**) Learning curves representing performance accuracy for various airflow pairs under different photoactivation conditions. (**G**) Learning curves representing animal accuracy for various odor-based tasks using same/different airflow rates (*P < 0.05).

Having observed the learning-dependent refinement of calcium activity in the inhibitory neurons during airflow discriminations, we further explored how the modulation of these neurons affects anemo-discriminations. We started by studying the anemo-discrimination behavior shown by heterozygous knockout mice where the deletion of the GluA2 subunit of AMPARs was specific to GAD65 interneurons (Fig. 5, A and B). Knocking out the GluA2 subunit from inhibitory granule cells of OB resulted in the enhancement of Ca^2+^ influx and synaptic inhibition, which facilitated odor discrimination (*9*). We trained one batch of mice to discriminate 0.5 LPM versus 0.4 LPM, and we observed a slower learning pace with the heterozygous GluA2 knockout mice compared to control mice (Fig. 5C, two-way ANOVA, *P < 0.05). This observation was in contrast to the effect of synaptic inhibition on odor discrimination behavior, which indicates that the extent of inhibition needed to refine the airflow information is different from that of the odor information. Furthermore, this genetic modulation was not restricted to the OB circuits. Therefore, we decided to characterize the phenotypes resulted by bidirectional optogenetic OB-specific modulation of GAD65 inhibitory interneurons.

The experimental animals consisted of different groups of mice expressing ChR2 and Arch in the GAD65 interneurons of the OB, while control animals had EYFP expressed in these interneurons. OB-specific modulation was achieved by implanting the light-emitting diode (LED) on top of the OB (Fig. 5D; see Materials and Methods). To identify the optimal photoactivation intensity, we studied mice’s airflow detection behavior in the presence of varying stimulation light intensities. For both ChR2- and Arch-expressing animals, the licking responses decreased as the intensity of photoactivation increased [Fig. 5E, (a) and (b)], whereas no change in the licking responses of control animals was observed [Fig. 5E(c)]. For further experiments, the light intensities were chosen in such a way that the licking responses of the animals during photoactivation remained similar to that under the no-photoactivation condition [Arch: range, 1 to 5 mW; mean, 2.93 mW; ChR2: range, 3 to 11 mW; mean, 6.96 mW; EYFP: mean, 6.96 mW (same for all animals)].

Furthermore, the role of inhibitory circuitry in controlling airflow discrimination efficiency was examined. Different groups of animals were trained on a series of airflow discrimination tasks, with alternating photoactivation ON and OFF conditions. For the first airflow pair (0.5 LPM versus 0.4 LPM, light OFF), animals reached the high-accuracy, asymptotic phase within 1200 trials. There was no difference in the learning pace across different groups, suggesting that intrinsic differences do not exist among the groups that can influence learning efficiency. However, when the animals were trained on 0.15 LPM versus 0.1 LPM under photoactivation conditions, we observed faster learning when GAD65-expressing interneurons were inhibited by Arch stimulation and slower learning during ChR2 stimulation (Fig. 5F, two-way ANOVA with Tukey’s multiple comparison test, *P < 0.05). A similar trend was observed for another airflow pair at 0.45 LPM versus 0.35 LPM under the photoactivation conditions. These bidirectional modulations in learning pace were specific to the photoactivation conditions, as no difference in learning pace was seen for another airflow pair (0.25 LPM versus 0.3 LPM) under no photoactivation (Fig. 5F, two-way ANOVA with Tukey’s multiple comparison test, *P < 0.05). On training the mice with the same airflow rate (0.6 LPM versus 0.6 LPM), animals’ performance remained at chance levels, confirming the specific learning of airflow discriminations (Fig. 5F). Photoactivation also altered the FDTs. ChR2-expressing animals showed slower FDTs when compared across different groups [fig. S8A(a)]. For all the animals performing above 80% across different airflow pairs under light-ON conditions, the Arch group showed significant improvement in FDTs as compared to control groups (fig. S9). However, this analysis could not be performed because the ChR2-expressing animals’ average performance accuracy was below 80%. These phenotypic differences did not arise as a result of varying sampling strategies that may occur because of optogenetic stimulations. In all groups, animals showed similar SFs across different airflow pairs [fig. S8B(a)], indicating that the alterations in airflow discrimination behavior were specifically due to the modulations of the OB inhibitory circuitry.

We continued to study the role of OB inhibitory circuitry in controlling odor discrimination. In contrast to airflow discriminations, opposing phenotypes were observed when the animals were trained on an odor discrimination task (Fig. 5G; octanol complex mixture: 60-40 versus 40-60 binary mixtures). The phenotype observed was consistent with previous findings where enhanced inhibition led to better odor discrimination abilities (*9*, *12*). The odor discrimination time (ODT) shown by the Arch group was significantly slower, and no difference in the SFs was observed between groups [fig. S8, A(b) and B(b)]. As contrasting phenotypes were observed for airflow- and odor-based discrimination tasks, we studied the changes in multimodal stimuli discrimination efficiency (odor A + airflow 1 versus odor B + airflow 2, 60-40 hexanal-pentanone + 0.4 LPM versus 40-60 hexanal-pentanone + 0.3 LPM) upon optogenetic modulation. All three groups of mice learned this discrimination with similar efficiencies. This indicates that the deficiencies we observed for either odor- or airflow-based discriminations under optogenetic modulations were rescued by altering the second modality added to the multimodal stimuli (Fig. 5G, two-way ANOVA with Tukey’s multiple comparison test, *P < 0.05). Similar DTs were observed for different groups. However, an increase in SF was observed for the ChR2 group [fig. S8, A(b) and B(b)]. Further experiments dissecting the connectivity between breathing and learning centers are needed to find out the reasons for this modulation.

Varying airflows associated with specific stimuli can cause concentration differences of odorants. Therefore, we varied the concentrations with a specific stimulus and challenged the animals to discriminate while bidirectionally modulating the inhibitory network activity. The phenotype during this concentration-based discrimination task was similar to that observed during the odor-based task; the ChR2 group learnt faster, whereas the Arch group showed learning deficits and higher ODT (Fig. 5G, two-way ANOVA with Tukey’s multiple comparison test, *P < 0.05). All groups of mice showed similar SFs during this task, while ODT for Arch animals was found to be slower [fig. S8, A(b) and B(b)]. Together, the bidirectional changes of behavioral readouts during the airflow and odor discrimination tasks and the rescue of the learning deficits during multimodal tasks under optogenetic modulations imply that synaptic inhibition in the OB refines the airflow and odor information processing. However, the optimal inhibition required for airflow- and odor-information refinement varies. Moreover, OB neural circuits might be playing a critical role in integrating the chemical and mechanical information and generating the ‘multimodal’ odor percept in the dynamic olfactory world.

### Mechanosensation through the mouse nose enhances olfactory perception under subthreshold stimuli conditions

**Fig. 6.**
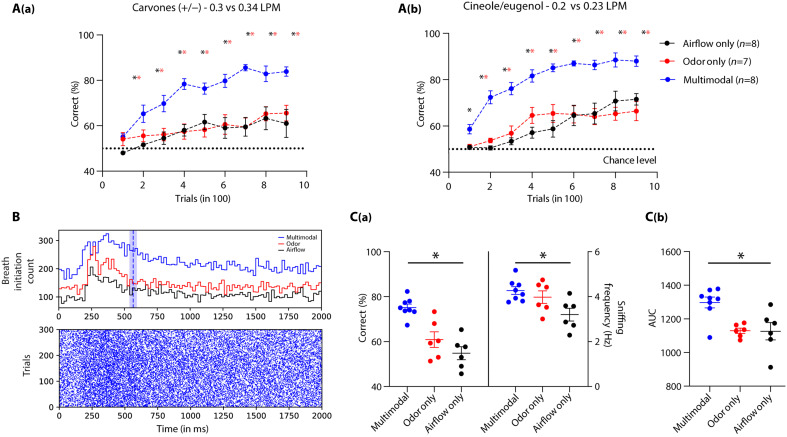
Multimodality generated by airflow-odor combination enhances discrimination learning at the subthreshold levels. (**A**) {[A(a)] and [A(b)]} Comparisons of learning curves of animals when trained to discriminate the unimodal stimuli (odors and airflows) and multimodal stimuli (odor + airflow). For different discrimination tasks, animals displayed enhanced learning while they were trained on multimodal stimuli (*P < 0.05). (**B**) Raster plots representing the inhalation onset for the multimodal discrimination task pooled across animals in the last phase of learning (last 300 trials). The histogram represents the pooled inhalation onset for odor and airflow only, as well as the multimodal group. The blue vertical line represents the DT for animals trained on multimodal stimuli, and the shaded region represents SEM. (**C**) [C(a)] Comparisons of accuracy (left *y* axis) and SF (right *y* axis) of animals when trained to discriminate the odor, airflow, and multimodal stimuli during the last task. The average accuracy and SF of animals during the last task for multimodal stimuli were significantly higher. [C(b)] Comparisons of the AUC calculated from the breathing of animals trained to discriminate the odor, airflow, and multimodal stimuli during the last task. The average breathing AUC of animals trained on multimodal stimuli was significantly higher in comparison to those trained on unimodal tasks (*P < 0.05).

In nature, rodents sample and perceive odorants carried by turbulent airflows. These odor plumes and the associated airflows make the olfactory environment very dynamic and may challenge animals to detect and perceive chemical cues even at subthreshold concentrations. Here, we explored the functional relevance of mechanosensation through the rodent nose in aiding olfactory perception under subthreshold conditions. To test this hypothesis, we trained different batches of mice on varying odor concentrations and airflow rates. To determine the minimum differences in airflow rates that mice can discriminate, they were trained on different airflow rates. Three flow pairs were identified as being below the discrimination threshold—0.1 LPM versus 0.11 LPM, 0.2 LPM versus 0.23 LPM, and 0.3 LPM versus 0.34 LPM (fig. S10A). These discrimination thresholds followed Weber’s law (fig. S10, B and C) (*36*, *37*). Furthermore, the discrimination thresholds were calculated for (+) carvone versus (−) carvone (0.01%, v/v) and cineol versus eugenol (0.01%, v/v; fig. S11). Next, we trained three different groups of mice on subthreshold discrimination levels for airflow rates and odors and on the “multimodal” stimuli combining both airflow rates and odors, i.e., (+) carvone versus (−) carvone (at 0.3 LPM versus 0.34 LPM) and cineol versus eugenol (at 0.2 LPM versus 0.23 LPM). Compared to odor-only and airflow-only groups, mice that were trained on “multimodal” stimuli showed a significantly enhanced learning pace [Fig. 6A, (a) and (b)]. Furthermore, to probe whether sampling differences could account for the observed results, another batch of mice was trained on similar stimuli under head-restrained conditions, and their sniffing behavior was quantified (Fig. 6B). The “multimodal” group showed high-performance levels of >80% at the end of training compared to other groups. Quantification of SF and inhalation onsets revealed enhanced breathing patterns under multimodal conditions compared to unimodal ones [Fig. 6C, (a) and (b)]. These results provide robust evidence that mechanosensation through the mouse olfactory system facilitates olfactory discrimination at subthreshold levels.

## DISCUSSION

The subsystems in the rodent nose make it a unique sensory organ by imparting the ability to detect chemical, mechanical, and thermal stimuli, yet the neural mechanisms underlying “multimodal” olfaction remain elusive (*38*). Our experimental results demonstrate that mice can discriminate various airflow rates with ~90% accuracy through their nose, even in the absence of whiskers, as confirmed by OSNs’ ablation and bulbectomy (Figs. 1 and 2). While our PID measurements did not reveal detectable changes in chemical composition across airflow conditions, it remains possible that mice are sensitive to trace-level volatile compounds below the detection threshold of the PID. To address this, we conducted control experiments using zero air, the stimulus directed to the nostrils and away from the nostrils, and the same airflow rates. The results of all these control experiments confirmed the learning of airflow rates used throughout the study. During anemo-discrimination decision-making, mice exhibited sniffing refinement, indicating the orthonasal airflow information processing (Fig. 3). Furthermore, the stimulus-dependent and learning-dependent calcium signals revealed the role of OB inhibitory circuits in processing airflow information (Fig. 4). Genetic/optogenetic modulations resulted in the bidirectional shift of anemo-discrimination learning pace, with contrasting phenotypes for olfactory learning, highlighting the role of optimal inhibition for refining odor and airflow stimuli (Fig. 5). Furthermore, enhancement in discrimination learning pace resulted from combining odor and mechanical stimuli at subthreshold levels, confirming the heightened olfactory perception aided by mechanical stimuli (Fig. 6). Thus, revealing the underlying neural mechanisms of mechanosensation through the olfactory system provides an unexplored dimensionality to the olfactory perception.

### Does the rodent olfactory system encode and refine airflow information?

Here, we report substantial differences between odor and airflow discrimination behavioral readouts. In a go/no-go paradigm used for investigating odor discriminations, we observed differences in the time taken by animals to discriminate simple and complex odors. The complexity of stimuli was quantified by assessing the overlap between evoked glomerular activity patterns (*9*, *13*, *35*, *39*, *40*). However, when we trained mice to discriminate airflow rates on a similar paradigm, no notable differences in the FDTs were observed across different airflow pairs. We altered the difficulty by varying the discriminable differences and the strength of airflow rates (Fig. 1D). Given the fact that reaction time measurements can provide the temporal limit of underlying neural mechanisms, these results may indicate a broader activation pattern evoked in the OB (*27*), potentially independent of stimuli complexity. A commonality we observed with the odor discrimination tasks is the modulation of learning pace and reaction times by the OB inhibitory network, however, notably in opposing directions. For example, the enhancement of inhibition caused a faster learning pace in odor discriminations (*12*), whereas it resulted in a slow pace for anemo-discriminations (Fig. 5, F and G). This may arise because of the differences in optimal inhibition required to refine the broader activation patterns evoked by airflows in the OB compared to odorants (*27*). The sniffing modulation observed during the airflow discrimination ratified the orthonasal airflow information processing (Fig. 3). The enhancement in sniffing toward the airflow compared to odor stimuli may indicate a strategy used by animals to have optimal activations needed for effective mechanosensation. However, whether the OB can encode and refine the airflow information remains an open question.

The c-Fos activation observed in the GABAergic interneuron population followed by airflow discrimination training prompted further investigation of Ca^2+^ dynamics. We observed a learning-dependent refinement in the interneuron activity (Fig. 4). As anemo-information processing by the OB circuits was never reported before, we adopted a multipronged experimental approach to investigate the causality. Deleting the GluA2 subunit of AMPARs that causes higher Ca^2+^ influx and enhanced inhibition (*9*) resulted in a slower learning pace, which was supported by the ChR2 stimulation experimental results as well. However, the Arch stimulation resulted in a faster learning pace, establishing the causality between inhibitory interneurons and anemo-discrimination (Fig. 5). Our study thus provides the first experimental evidence supporting the encoding and refinement of airflow information by the rodent olfactory system, an idea that existed for the past 70 years (*1*, *2*).

### Mechanosensation at the peripheral olfactory system

Approximately 50% of OSNs exhibit mechanical sensitivity, as studied under in vitro conditions, where the odor receptors (ORs) have been proposed as the sensors (*5*, *6*). Many G protein (heterotrimeric guanine nucleotide–binding protein)–coupled receptors have been shown to be multimodal sensors executing different functions (*41*–*43*). The loss of mechanosensitivity upon treatment with adenylyl cyclase inhibitors or the knockout of the cyclic nucleotide–gated channel CNGA2 suggests that the second-messenger pathway, essential for odorant processing, also mediates mechanosensation (*6*). Moreover, OSNs that express different ORs showed variable sensitivity toward mechanosensation (*5*). All these observations point to a plausible sensing mechanism by ORs.

The deformation of the neuronal membrane by mechanical stimuli evoked by sniffing modulations and aerodynamics in the nasal cavity may result in enough conformational changes in mechanosensitive ORs, which ultimately lead to the second messenger cascades. However, in our experiments, we observed a complete shutdown of mice’s ability to discriminate airflows after ZnSO_4_ and methimazole treatments. If only 50% of OSNs show mechanical sensitivity, how would partial OSN ablation (Fig. 2C) lead to a complete loss of anemo-discrimination capability? It is possible that additional mechanosensors are present in the olfactory epithelium, which are yet to be found. Earlier experiments in the tracheotomized rats proved that the OB neurons fire in accordance with the modulations of airflow through the nasal cavity (equivalent for sniffing) while the animals breathe at a basal rate (*44*). This supports the notion of respiratory modulation acting as reafferent signaling in the perception. The mechanosensation through the OSNs may form the basis for this reafferent signaling.

### OB inhibitory circuits refining the anemo-information processing, a previously unknown function of the mouse olfactory system

The enhanced sniffing observed during airflow discrimination likely increases the spiking activity of mechanosensitive neurons. In complex olfactory environments, where animals are challenged to detect and discriminate volatile compounds from odor plumes, this additional channel of information can increase the sensitivity of the olfactory system. This was evident in our experiments, where we observed a significant increase in the learning pace of animals along with heightened sniffing when two subthreshold stimuli (odor and airflow) were combined (Fig. 6). Rhythmic activity has been reported in the OB neurons even in the absence of odor molecules (*4*). Is this activity driven solely by mechanosensitive neurons at the periphery, or are there mechanosensors in the OB? In a recent article, the authors report local field oscillations evoked by vascular pressure pulsations. This was mediated by mechanosensitive ion channels present in a subset of OB projection neurons (*45*). These observations lead to the fundamental question: Can OB circuits process and refine the mechanical stimuli? If so, how?

The ethmoidal branch of trigeminal nerve innervates the main olfactory epithelium and the OB. Despite the limited innervation, it can modulate the perception of certain odorants (*46*–*48*). However, we see a huge activation in the OB inhibitory interneuron layer in response to airflow stimuli (Fig. 4). Moreover, the OB interneuron–specific optogenetic modulation resulted in robust phenotypes (Fig. 5). Therefore, we conclude that the airflow detection and discrimination we report here are mostly carried out by the main olfactory system.

Odor encoding by OB projection neurons’ temporal patterns of spiking is very well established (*19*, *20*, *49*–*54*). Furthermore, this activity can be driven by nasal respiration (*20*, *55*, *56*). Sniffing modes used by animals can help them to capture olfactory “snapshots” (*16*, *20*, *57*, *58*). The glomerular-specific oscillatory activity recorded from mitral and/or tufted cells was proposed to be caused by the mechanosensory channel. The phase of these oscillations remained unchanged across different airflow rates. However, the introduction of odors caused the phase shifts, which were lost in the absence of mechanical stimuli, implying the role of mechanosensation in the phase coding of odor information (*26*). Disruption of OB inhibition altered spike phasing within the respiration cycle, ultimately impairing odor discrimination (*59*). These observations indicate a probable correlation between OB inhibition and mechanosensory information processing if OB circuits are involved. Given all these factors, we modulated the synaptic inhibition in different ways. Deleting the GluA2 subunit of AMPARs or optogenetically activating GAD65-expressing neurons, the major class of inhibitory interneurons (*60*) that causes the enhancement of inhibition, resulted in compromised anemo-discrimination learning. However, inhibiting these GABAergic neurons improved the learning. Moreover, animals showed contrasting learning phenotypes for odor discriminations with these modulations, setting the optimal inhibition needed for the refinement of odor and airflow information (Fig. 5). Considering the heterogeneous interneuron population in the OB (*61*, *62*) and their potential involvement in various disease conditions (*63*, *64*), it is essential to probe “multimodal” olfaction in smell disorders as well as in the animal models for these conditions (*65*–*73*).

Learning-dependent refinement of sniffing behavior was observed during odor discriminations (*35*) and anemo-discriminations (Fig. 3). Mechanosensory signals facilitate the phase coding of odors in the OB. It appears to be driven more by the sniffing rhythm than by the amplitude. However, the increase in nasal airflow rate enhanced the response amplitudes in several glomeruli without affecting the phase (*26*, *54*). Our data suggest that differences in the ambient airflow strength can modulate these response amplitudes correspondingly, which can facilitate the discrimination between various airflow rates. Under subthreshold conditions, introducing odors will bring changes in the phase shifts, whereas the airflow rate differences would result in the modulation of response amplitudes. Therefore, a combination of phase and rate coding at subthreshold levels will help animals integrate both chemical and mechanical cues to enhance odor perception. We believe that these results suggest a potential model by which ambient airflow influences mechanosensation through orthonasal respiration and, therefore, multimodal olfactory perception. Therefore, our findings provide a conceptual framework for future research to delineate the underlying mechanisms.

**Fig. 7.**
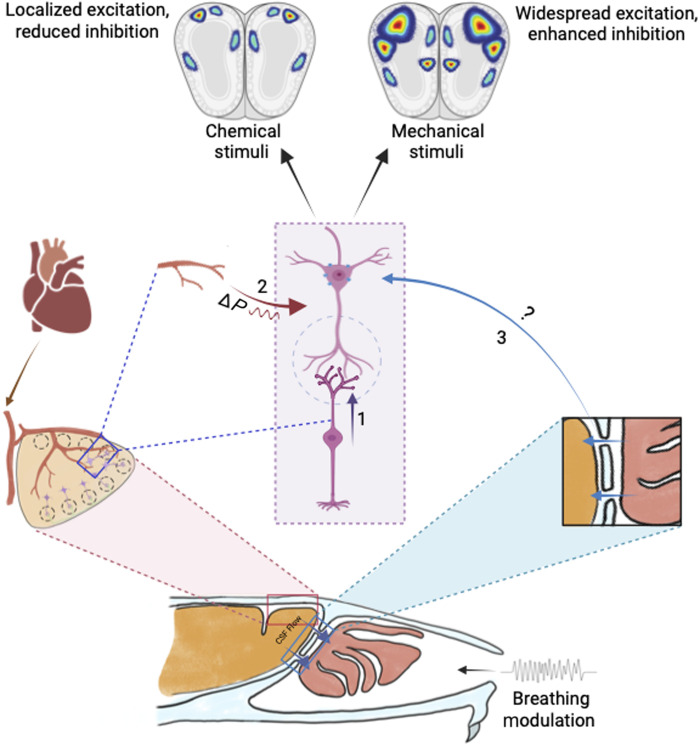
Proposed model of the differential recruitment of inhibitory network for the chemical and mechanical information processing in the mouse OB. Mechanosensation through the OSNs can excite projection neurons of the OB (marked as 1 in the figure) (*5*, *6*). Vascular pressure pulsations also modulate the neuronal activity in the OB (marked as 2 in the figure) (*45*). The role of orthonasal respiration in modulating the intracranial pressure changes transduced from the nasal cavity to the cranial cavity remains an open question (marked as 3 in the figure) (*79*). Together, vascular pulsations and respiratory-driven pressure changes may contribute to intracranial pressure modulation, leading to widespread excitation and enhanced inhibition in the OB in response to mechanical stimuli compared to odor stimuli. Created in BioRender. N. M. ABRAHAM (2025); https://biorender.com/4muxh8v.

Can these effects be achieved solely by the mechanosensory drive through the OSNs? Or do we have to probe beyond the sensory periphery? Recent articles (*45*, *74*, *75*), along with our analysis using FM 1-43 dye uptake, which has been shown to be Piezo2 activity–dependent, confirm the presence of mechanosensitive ion channels in a subset of OB projection neurons (fig. S12) (*76*–*78*). Vascular pressure pulsations can modulate the spiking activity of mitral cells in the OB through mechanosensitive channels (*45*). Given the continuity of cerebrospinal fluid between the cranial and nasal cavities, pressure pulsations generated in the nasal cavity through the respiratory modulations can be transduced to the cranial cavity (fig. S6) (*79*). How these modulations affect the airflow detection and discrimination abilities remains an open question. Both these modulatory pathways can result in enhanced excitation in the OB in response to mechanical stimuli compared to odor stimuli (Fig. 7). These differences can be attributed to the varying optimal inhibition needed to refine mechanical and chemical stimuli in the OB and the opposing phenotypes observed when the inhibitory network is optogenetically stimulated (Fig. 5). The previously unknown function of the olfactory system we report here may help reveal the mechanisms of improved perception and cognition caused by integrative body-mind training where better physiological parameters of heart rate and breathing are observed (*80*).

## MATERIALS AND METHODS

### Animals used

A total of 192 normally weaned C57BL/6J males (6 to 10 weeks at the beginning of the experiments) was used for all the experiments (experiment-wise breakup of animals is mentioned in the corresponding data). In addition to the wild-type animals, transgenic mice were generated by crossing the following genotypes that were obtained from the Jackson Laboratory, unless mentioned otherwise: GluA2^2Lox^: GluA2^2Lox^ (Gria2) was obtained from the Heidelberg University, Germany; GluA2^GAD65(+/−)^ knockout: GluA2^2Lox^ with B6N.Cg-Gad2^tm2(cre)Zjh^/J for the knockout of GluA2 in GAD65 + ve neurons; GAD65-EYFP: B6N.Cg-Gad2^tm2(cre)Zjh^/J with B6.129X1-Gt(ROSA)26Sor^tm1(EYFP)Cos^/J for the expression of EYFP in GAD65 + ve cells; GAD65-GCaMP6f: B6N.Cg-Gad2^tm2(cre)Zjh^/J with B6;129S-Gt(ROSA)26Sor^tm95.1(CAGGCaMP6f)Hze^/J for the expression of GCaMP6f in GAD65 + ve cells; GAD65-Arch: B6N.Cg-Gad2^tm2(cre)Zjh^/J with B6;129S-Gt(ROSA)26Sor^tm35.1(CAGaop3/GFP)Hze^/J for the expression of Arch in GAD65 + ve cells; GAD65-ChR2: B6N.Cg-Gad2^tm2(cre)Zjh^/J with B6;129S-Gt(ROSA)26Sor^tm32(CAGCOP4*H134R/EYFP)Hze^/J for the expression of ChR2 in GAD65 + ve cells. A total of 23 GluA2^2Lox^ (5 for Western blotting and 18 for discrimination learning), 22 GluA2^GAD65(+/−)^ knockout (6 for Western blotting and 16 for discrimination learning), 26 GAD65-EYFP (12 for c-Fos staining and 14 for optogenetics), 16 GAD65-GCaMP6f (calcium imaging), 15 GAD65-Arch (optogenetics), and 10 GAD65-ChR2 (optogenetics) animals was used in different experiments.

### Ethical approval

The experimental procedures used in this manuscript are approved by the Institutional Animal Ethics Committee (IAEC) at the Indian Institute of Science Education and Research (IISER) Pune and the Committee for the Control and Supervision of Experiments on Animals (CCSEA), Government of India (animal facility CCSEA registration number 1496/GO/ReBi/S/11/CCSEA). The usage of animals in this manuscript was approved under protocol numbers IISER/IAEC/2017-02-006 and IISER/IAEC/2017-02-008.

### Maintenance of animals used in the study

All experiments were conducted on adult male mice. The age of mice at the beginning of the experiment was 6 to 8 weeks. Mice were housed in individually ventilated cages in a temperature- and humidity-controlled animal facility on a 12-hour light-dark cycle. The mice were given standard rodent bedding and nesting material. All the experiments were performed during the light cycle. On the days of behavioral training, mice were fed ad libitum food but subjected to a water restriction schedule, designed to keep them at >80% of their original body weight. The water restriction schedule was never more than 12 hours long.

### Surgical procedures

#### Whisker trimming

Animals were mildly anesthetized with isoflurane, and whiskers were trimmed in a row-wise fashion using scissors. Whisker trimming was performed once a week on the experimental animals. Animals that were on a water restriction schedule were given free water for 2 hours before and after the treatment.

#### Whisker plucking

The process of whisker plucking was identical to that of whisker trimming. Instead of clipping the whiskers, tweezers were used to pluck them from the root.

#### Intranasal ZnSO_4_ injections

Animals were mildly anesthetized with isoflurane and were held with their snout facing upward. Using a micropipette, 25 μl of 5% ZnSO_4_ was infused into one of the nostrils. The animal was turned upside down immediately after the infusion to reduce the passage of ZnSO_4_ into the windpipe or lungs. Before infusing the second nostril, the animals were given a 2-hour recovery period. After infusing both nostrils, the animals were given a 2-day recovery time with ad libitum access to food and water. Animals that were on a water restriction schedule were given free access to water for 12 hours before the treatment.

#### Methimazole injection

Methimazole (75 mg/kg) dissolved in PBS was injected intraperitoneally (IP injection). Animals were given a two-day recovery period following injections. Animals on a water restriction schedule were given free access to water for 12 hours before the treatment.

#### Surgical aspiration of OB (bulbectomy)

The animals were anesthetized with a combination of ketamine and xylazine. The anesthetized animal was then mounted on a stereotaxic instrument. An incision was performed on the skull over the OB area, and the skull was completely cleaned. A 2.5-mm-diameter cranial window was formed over the OB region using a biopsy punch. A 21-gauge needle connected to a vacuum pump was used to aspirate the OB. The skin was sutured back together, and the animal was given a 2-week recovery time before continuing with the experiments. All of the steps were the same for the animals that had sham surgery, except for the excision of the OB.

#### Head post implantation for head-restrained behavioral training

The head post was surgically implanted on top of the animal’s skull. First, the animal was anesthetized with an intraperitoneal injection of ketamine (50 g/g body weight) and xylazine (10 g/g body weight) and was mounted on the stereotaxic instrument. A local anesthetic gel (lignocaine hydrochloride) was applied to the skin over the skull, and an incision was made. Using a cotton swab, the region was thoroughly cleaned with artificial cerebrospinal fluid (125 mM NaCl, 5 mM KCl, 10 mM glucose, 10 mM Hepes, 2 mM CaCl_2_, and 2 mM MgSO_4_ in 1 liter of sterile distilled water, pH 7.4). The periosteum of the skull was carefully scraped off with a scalpel blade. The skull was allowed to air dry, and an etching agent was applied to the exposed skull for 15 s. A liquid primer was then applied to the dried skull using the applicator, and the primer was allowed to air dry for 10 s, followed by ultraviolet (UV) light exposure for 30 s. The primer serves as an adhesive for the UV-polymerizing cement to adhere to the bone.

A very thin layer of UV cure dental cement was spread on the skull and polymerized using UV light for 20 to 30 s, on which the head post was mounted and exposed to UV for curing. The remaining part of the skull was filled with cold cure acrylic dental cement. The mouse was unmounted from the stereotaxic instrument and placed in the home cage. The animal was given a recovery period of 2 days.

#### Cranial window and LED implantation for optogenetics

Blue or amber LEDs were implanted over the OB region to achieve the optogenetic modulation of OB-specific GAD65-expressing interneurons. To avoid direct contact with brain tissue, the LEDs were implanted over cranial windows. The animals were first anesthetized with a combination of ketamine and xylazine and were placed on the stereotaxic instrument. An incision was made over the skull, and the skull was thoroughly cleaned. A 2.5-mm-diameter window was created over the OB region using a biopsy punch, which was then covered by a glass coverslip by sealing its side with cold cure dental cement. On the skull, a head post was implanted posterior to the cranial window. The clarity of the cranial window was checked after a week, and an LED was mounted over it with cold cure dental cement.

#### Implantation of imaging cannula for in vivo calcium imaging

The animals were anesthetized with a combination of ketamine and xylazine, and a 1-mm-diameter cranial window was created in the center of the right hemisphere of the OB using a dental drill. As a precaution, a blunt Hamilton needle was lowered 1 mm ventral to the dura in the OB and kept as such for 5 min to create a path before the insertion of the GRIN lens. A value of 1 mm was chosen on the basis of the observation that most of the GAD65-expressing interneurons lie at this depth. The 1-mm protruding GRIN lens was lowered vertically until it reached the GCL. To secure the lens assembly to the skull surface, a combination of cyanoacrylate gum and acrylic dental cement was used. A head post was implanted behind the implanted lens, and animals were given a month to recover.

#### Implantation of cannula for pressure recording inside the nasal cavity

The breathing of the animals was recorded from the nasal cavity, as described previously (*22*, *81*). Briefly, the animals were anesthetized with a combination of ketamine and xylazine, and around 1-mm-diameter cranial window was created in the right nasal cavity using a dental drill. A 7-mm-long 21-gauge cannula was implanted in the nasal cavity. The recordings were carried out under awake conditions. The cannula and pressure sensor (MPX5050) were connected through polyethylene tubing. The signal was filtered through a bandpass filter, and data acquisition was made using a Picoscope.

##### Naïve mice

The data were collected in the form of ac signals. During experiments, animals were administered with different airflow conditions (100, 200, 300, and 400 ml/min) in a pseudorandomized order. Data were collected for two batches of animals. For the first batch of animals (*n* = 6), data were collected from 20 trials per airflow in awake animals using a Picoscope with a time resolution of 0.5 ms. For the second batch (*n* = 5), the number of trials was increased to 40. Different parameters such as relative inhalation amplitude, exhalation amplitude, and frequency were calculated. Pearson correlations were calculated both for individual animals and for combined data across all animals.

##### Trained mice

We trained a group of mice (*n* = 8) for two different airflow pairs (0.1 LPM versus 0.2 LPM and 0.3 LPM versus 0.4 LPM) under the head-restrained condition until they learned to discriminate with high accuracy. Breathing data were recorded for each airflow condition for 40 trials. Breathing parameters similar to those assessed in naïve animals were then quantified and compared.

##### Analysis

1) For animal-specific correlations, parameters were normalized to the corresponding average value at 100 ml/min airflow. For example, if an animal had 40 trials per airflow, the average amplitude for 100 ml/min was calculated, and amplitudes for other airflows (100, 200, 300, and 400 ml/min) were normalized to this value. This was done for all parameters.

2) For pooled correlations, average values for each parameter at each airflow were first calculated per animal and then normalized to the corresponding value at 100 ml/min. For instance, if the average inhalation amplitude for animal M01 at 100 ml/min was *x*, the amplitudes for all the airflows were normalized relative to *x*, making the relative amplitude 1 for 100 ml/min. This normalization was performed for all animals, and the correlation was then computed.

#### Stereotaxic injection of FM 1-43 dye

FM 1-43 dye that specifically labels Piezo2 activity was injected in the OB of the wild-type mice using the stereotaxic instrument (*77*). The animal was first anesthetized with an intraperitoneal injection of ketamine (50 g/g body weight) and xylazine (10 g/g body weight) before being mounted on the stereotaxic instrument. A ~2.5-mm-diameter cranial window was made above the OB. The region was thoroughly cleaned using artificial cerebrospinal fluid. The intersection of the inferior cerebral vein with the hemispheric midline and bregma was put in the same plane. Twelve to fourteen injections were administered in each OB at different locations specifically targeting the dorsal and lateral mitral cell layers in each location. Approximately 100 to 150 nl of FM 1-43 dye (Invitrogen, T3163, diluted in PBS) was injected in each location. The FM 1-43 dye intake was imaged by imaging the fluorescence emission of the dye at ~580 nm.

### Operant conditioning procedures

#### Behavioral training under freely moving conditions

##### Apparatus

Custom-modified eight-channel olfactometers (from Knosys, Washington) (*82*) were used for airflow/olfactory discrimination experiments (fig. S1A). The instruments were controlled by a customized program written in IGOR (Wavemetrics). The device included an operant chamber where the animals were placed for behavioral training. A circular sampling/reward port secured by an infrared (IR) beam was located on the right side of the operant chamber. Animals could start a trial by inserting their head and breaking the IR beam that guarded the sampling/reward port. Upon trial initiation, stimuli (either airflow or odorants) were delivered via an airflow pump connected to flowmeters and solenoid valves. When a trial is initiated, a set of solenoid valves opens, allowing air to pass through the flowmeter and reach the animal. The precise onset of the stimulation was assured by a system of solenoid valves controlled by software. Airflow strength (rates) was regulated by calibrated flowmeters, standardized to deliver specific rates. Standardization was achieved using a hot-wire anemometer (model: PCE-423-ICA, PCE Instruments). For airflow discrimination training, to minimize the possibility of animals using residual odors as cues, the air was passed through activated charcoal. In addition, separate experiments were conducted using air from a zero-air cylinder, delivering airflows at the nostrils and caudal to the nostrils and the same airflow rates. In addition, three different modes of stimulus delivery were used on the basis of the nature of the behavioral experiment (Fig. 1 and fig. S1).

In mode 1, the stimulus was delivered on the snout of animal from the top (Fig. 1A). The stimulus was delivered by a 4-mm-diameter circular tube. The distance between the delivery tube and the snout was kept fixed at around 8 to 10 mm. The input airflow rate supplied and the actual output rate observed for mode M1 were quantified and found similar (fig. S1A).

In mode 2, the stimulus delivery was targeted on the whiskers. Two protruding tubes (4 mm in diameter) delivered focused stimulation to both sides of the snout [Fig. 1E(a)]. The delivery tubes protruded sufficiently to be in the same plane as the lick tube. When the animals poked their heads into the sampling port, they were provided with the airflow stimulus on their whiskers. The radial distance between the lick port and each tube was 8 mm. The delivered input airflow rate and the observed output rate for mode M2 output tubes were quantified and found to be equivalent (fig. S1B).

In mode 3, the stimulus delivery was targeted on the whiskers but in a diffused way. The stimulus was provided to the whiskers by four holes (4 mm in diameter each) arranged in a semiarc around the lick tube [Fig. 1E(b)]. In this mode, the plane of the lick tube was protruded in comparison to that of the delivery ports. When mice poked their head in the sampling port, they received the stimulus in a diffusive manner. The radial distance from the center of the lick port to that of each hole was also 8 mm in this mode. The real output rate for different output holes of mode M3 was quantified, and the actual observed output was found to be as expected (fig. S1C). For modes M2 and M3, the airflow rates mentioned during the discrimination training are the total output rates from either side, i.e., 0.6 LPM means that animals received 0.6 LPM from both sides of the snout.

##### Task habituation phase

Three or four days after the start of water deprivation schedule, animals underwent a task habituation training. Animals were trained using standard operant conditioning procedures (*40*). The task habituation was completed in nine phases. The animals received a water reward (2 to 3 μl) simply by breaking the IR beam in the first pretraining phase (phase 0). This enabled the animals to locate the reward port and lick tube. In the next stage, the animals only receive water when they register at least a single lick. After 15 such trials, the subsequent phase of task habituation begins, wherein the trial is initiated only when the IR beam is broken by a nose poke. The beam break causes the instrument valves to open, resulting in stimulus presentation for 2 s. The task’s complexity steadily increased from this stage onward to train the mouse to lick continuously during stimulus presentation. Airflows with precise flow rate and diluted odors were delivered for the airflow- and odor-based pretraining tasks, respectively. Unless specified otherwise, odors with a concentration of 1% were used and diluted with mineral oil. All animals completed the pretraining in three or four sessions of 30 min.

##### Discrimination training

All the airflow- and odor-based discrimination training was performed on a go/no-go behavioral paradigm (*40*, *82*). The mouse initiated a trial by breaking an IR beam that guards the sampling port (Fig. 1A). This enabled the opening of one of the solenoid valves, followed by the opening of a three-way diversion valve after 500 ms. After the diversion valve is opened, the stimulus is presented to the animal for a specified duration. The use of a diversion valve reduced the period between the onset of the stimulus and the first contact with the animal. To obtain a reward, the animal has to meet the required reward criteria based on the reward contingency of the stimulus [rewarded (S+)/nonrewarded (S−)].

The response time provided to the animal was virtually divided into four equal bins. (For example, if the given response duration is 2 s, it will be virtually divided into four 500-ms bins.) The response time was generally kept the same as the stimulus duration. For an S+ trial to be correct, the animal had to register a lick in at least three of these four bins. For a correct S+ trial, the animal received a water reward of 3 to 4 μl after the end of the stimulus (reward criterion: the animal needs to register a lick in at least three out of four bins). To be successful in an S− trial, the animal should not lick in more than two bins. A subsequent trial did not begin unless an ITI of 5 s had passed between trials. The set ITI was long enough for the mouse to retract fast at the end of a trial. There were no regulations in place to compel the mouse to sample the stimulus for a minimum time before making a decision. Furthermore, there were no rules in place to restrict licking during the prestimulus time.

Stimuli were delivered to the animals in blocks of 20 trials. Each of these blocks contained 10 S+ and 10 S− trials. The S+ and S− trials within a block were pseudorandomized so that no more than two stimuli of the same reward contingency were delivered consecutively. The rewarded stimuli were always counterbalanced with the nonrewarded stimuli during the training phase, i.e., in a group of experimental animals, half of them were trained with higher airflow as the rewarded one, and the other half were trained with lower airflow as the rewarded cue to mitigate any potential bias toward a particular stimulus. Animals were sufficiently motivated to complete 200 to 300 trials each day, spread out over one to two (20 to 30 min) sessions. Usually, the performance accuracies of animals were calculated by averaging 100 trials (five blocks of 20 trials). Different instrumental readouts, such as ITI and licking frequency, were used to track the motivation of animals. When the animal ceased licking for the rewarded trials, the training session was stopped.

##### Data acquisition

The data were collected using custom-written software in IGOR-PRO that was compatible with the MCC-CIO-DIO 48 data acquisition card.

##### Video recording of animals

The videos of the animals performing the airflow discrimination task under freely moving conditions were captured with a Redmi 4A smartphone at a resolution of 30 frames per second.

##### Novel object recognition task (NORT)

A total of six GAD65-EYFP mice was used for the experiment. Animals were handled twice a day for 2 days before starting the experiment. The experimental setup consisted of a square box with dimensions of 40 by 40 by 40 cm. Two habituation sessions, each lasting 5 min, were conducted to acclimate the mice to the arena. For experiments, two sets of similar objects—a transparent glass bottle and a Pyraminx cube—were used. The objects were counterbalanced within the groups to eliminate any potential bias toward a particular object type. The third day was the familiarization day, which was followed by a testing phase 1.5 hours later. During familiarization, either two glass bottles or Pyraminx cubes were kept diagonally slightly away from the corners, and mice were allowed to explore the objects for 10 min. In the testing phase, one of the objects was replaced with a novel object. For instance, in one group, a glass bottle was replaced with a Pyraminx cube, and in the other group, a Pyraminx cube was replaced with a glass bottle. Mice exhibited active sniffing for the maximum duration of testing toward the new object.

#### Behavioral training under head-restrained conditions

##### Apparatus

The head-restrained apparatus was custom built to deliver airflow/odor as the stimulus. A lickometer, a 10-channel olfactometer, a stimulus delivery nozzle, a respiration/breathing sensor, a lick tube, and a polyvinyl chloride tube were all part of the setup. The lickometer serves as an interface between all of the individual components that record and digitize the lick and breath responses of the animal to various stimuli. The olfactometer was equipped with 11 mass flow controllers and electromagnetic solenoid valves, enabling the experimenter to attain temporal precision during odor delivery. A splitter put in the olfactometer divided a clean air stream and routed it to various mass flow controllers. Air entered odor bottles via 10 small controllers, while air from the main controller was regulated by solenoid valves and used as a dilution air stream. The dilution and odorized air streams were combined at the output through a T-tube before entering the odor delivery nozzle. The stimulus was delivered to the nostrils of the restrained animal by the nozzle. A preloading duration was set before the odor presentation to guarantee that the odor plume reached a stable state. After the preloading time, the exhaust was turned off, allowing the stimulus to be delivered to the animal. This enabled us to obtain a homogeneous stimulus presentation. The equipment consisted of a polyvinyl chloride tube in which the animal was placed, and the head was restrained by screwing the head post onto a custom-built metallic plate attached to the tube. A lick tube was positioned near the animal’s mouth to record the animal’s lick state during the trial. On finishing the trial and completing the reward criteria, the animal received a water reward from the lick tube. The sniffing behavior of mice performing odor-based decision-making tasks under head-restrained conditions was noninvasively monitored using a thermocouple-based pressure sensor placed near one nostril. The instrument was controlled by a LabVIEW application that was custom written. During the head-restrained training, the ITI was kept constant at 13.2 s as this was the ideal intertrial period we observed while training animals under freely moving conditions.

##### Task habituation phase

Animals were subjected to task habituation training 3 to 4 days after the start of the water deprivation schedule. This was done to ensure that the animals became acclimated to the instrument and the procedures associated with it. Standard operant conditioning approaches were used to train the animals. This task habituation phase was completed in stages. Regardless of the animals’ responses during the first stage, they got a water reward of 3 μl after the presentation of a 200-ms short tone. This was done for 20 trials, after which the delay between tone and reward was raised to 1 s (20 trials) and then 2 s (20 trials). This was done to elicit licking behavior in water-deprived animals. Once the animals had learned to wait for the water, a stimulus was given to them. The stimulation lasted 2 s and came before the reward. The stimulus was also delivered to the animal during the next stages of the task habituation phase, and they had to learn to lick solely during the stimulus delivery duration. If animals only licked when a stimulus was present, they received a water reward after meeting the reward criteria (phase 1: minimum 80 ms of licking and the lick is registered in at least one of four virtually segregated bins; phase 2: 120 ms, two of four bins; phase 2.1: 240 ms, three of four bins). If animals licked before the onset of the stimulus, the required licking time to obtain the reward was increased to 200% of the abovementioned criteria. All of the animals completed the pretraining in four or five sessions of 30 to 40 min each. When mice completed the training, they learned not to lick during the baseline or time before the stimulus onset.

##### Discrimination training phase

Using a go/no-go behavioral paradigm (*35*, *39*), animals were trained to discriminate between two different stimuli, one of which was rewarded (S+) and the other was unrewarded (S−). The discrimination task was performed with a constant ITI of 13.2 s. The start of a trial causes one of the valves that control the stimulus to open. The stimulus was deflected to the exhaust for the first 1 s using a vacuum pump linked to the glass funnel. This was done to mitigate the time difference between the stimulus onset and its encounter with the animal. During this 1 s, the baseline licking was also assessed. The stimulus was presented for 2 s, and animals had to respond within this time frame to get a reward. The time required to lick to receive the reward for an S+ trial was determined by the baseline licking. If animals did not lick during the baseline, they were required to lick for a total of 240 ms in three (minimum 80 ms in each bin) of four 500-ms time bins. If the animals licked at the baseline, they had to lick twice as long during the stimulus presentation to get the reward and register it as a correct trial. For an S− trial to be correct, the licking of the animal should not exceed a total of 80 ms, regardless of whether it displayed baseline licking or not. There was no punishment for the incorrect trials. Training was carried out as explained under freely moving conditions. The frequency of licking was used to track the motivation of animals. When the animals were not motivated, they ceased licking for the rewarded trials.

### Measurement of breathing parameters

The breathing dynamics of the animal was continuously recorded during behavioral training using a noninvasive airflow pressure sensor placed near one of the nostrils. The sensor delivered real-time analog signals to the lickometer-linked breathing circuit with a resolution of as good as 4 μS. Using a threshold function, the lickometer breathing circuit converted the raw analog signals to a digital signal. The thresholding transformed the raw signal into binary values of 0 and 1, with a 0-1 transition representing an inhalation and a 1-0 transition representing an exhalation. On a trial-by-trial basis, these signals were continuously updated in the results file.

### Data acquisition

Data were collected using a custom-written LabVIEW program that was compatible with a National Instruments (PCI 6703) data acquisition board.

### Behavioral readouts

#### Learning curve

We used a measure known as the learning curve to visualize the learning pace and its magnitude. The learning curve measures the change in the percentage of correct responses as training progresses. Each point on the learning curve indicates the average accuracy of 100 trails [50 S+ and 50 S−] across all animals.

#### Discrimination time

The time point when the licking response of the animals toward S+ and S− stimuli considerably diverges after the stimulus onset is referred to as the DT. The licking behavior of each mouse was monitored to assess the DT. Animal licking behavior was recorded with a high temporal resolution and analyzed in time bins of 20 and 2 ms for freely moving and head-restrained conditions, respectively. The licking behavior changed as a result of learning and was considerably different between the beginning and the end of learning. Early in their learning process, when the animals could not discriminate the difference between S+ and S− stimuli, they licked for both stimuli. As a result, during the initial training phase, the animal’s lick responses to S+ and S− stimuli were comparable. However, as soon as they were able to differentiate between two stimuli, they began to selectively lick for S+ trials and avoid licking for S− trials, which caused a divergence in the lick responses between the two stimuli. The DT was determined using a sigmoidal curve that represented the difference between the S+ and S− licking responses. The DT was measured task-wise, i.e., for 300 trials. The statistical comparison of the lick responses between the S+ (150 trials) and S− (150 trials) trials yielded a *P* value curve as a function of time. The DT was considered as the last time point where the *P* value was less than 0.05.

#### Intertrial interval (ITI)

The ITI is the time that an animal takes between two consecutive trials. ITIs were used as the proxy for the motivation level of the animals. Under freely moving conditions, ITIs were variable and depended upon the animals, whereas for head-restrained behavior, ITI was kept fixed at 13.2 s. For head-restrained training, the motivation is monitored on the basis of the lick behavior of the animals.

#### Breathing analysis

The breathing frequency was measured for each trial throughout the stimulus time as well as before, during, and after the decision-making window. Assuming that the discriminating time is *x* ms, different windows were defined as follows: pre-DT from 0 − *x* to 0, during DT from 0 to 0 + *x*, and post-DT from 13,000 − *x* to 13,000. The onset of inhalation time points represented a point in the raster plot. A histogram with a bin resolution of 20 ms was used to depict the inhalation onset distribution across all trials and animals. The time of the first inhalation after the onset of the stimulus was considered as the inhalation onset time. The AUC was determined using the trial-wise histograms plotted for each animal. The AUC was computed just from the start of the stimulus to the DT/stimulus time. The sniffing peak latency was defined as the time corresponding to the mode of distribution of breath initiations. For analysis, custom-built algorithms in Python were used.

### Immunohistochemistry

#### Hematoxylin and eosin staining

The procedure for hematoxylin and eosin staining was adopted from our previous study (*70*). Briefly, the mice were perfused with 1× PBS and 4% paraformaldehyde (PFA), and the nasal cavity was dissected and kept for a week in a 10% EDTA solution to decalcify the bones surrounding the cavity. Then, the nasal cavity was embedded in paraffin. Using a microtome (RM2235, Leica Biosystems), 5- to 8-μm-thick sections were obtained and transferred to the poly-l-lysine–coated slides, and the tissues were stained with hematoxylin and eosin, as described previously (*70*). The DPX mounting medium was added to the slides, they were mounted with a coverslip, and the edges of the coverslip were sealed. A bright-field microscope (BX43, Olympus) was next used to examine the sections and capture the images. For each animal, two or three regions of interest (ROIs) per section were imaged depending on the quality of the sections from 8 to 10 sections per animal. The thickness of the olfactory epithelium (OE) was measured as the maximum perpendicular length from the basal membrane and compared across different groups. The thickness of the OE was used as a proxy to quantify the effect of different treatments on the olfactory epithelium (*33*, *34*).

#### Pizeo2 staining

The mice were transcardially perused using 1× PBS and 4% PFA, and the brain was dissected and stored in PFA at 4°C for 24 hours. The brains were then cryopreserved in a 30% sucrose solution for 24 to 48 hours. Coronal sections (50 μm thick) were taken using a Cryotome (Thermo Fisher Scientific) and were collected in a 24-well plate containing 1× PBS. The sections were then washed thrice for 5 min each with PBST (PBS with 0.1% Triton X-100). Nonspecific sites were blocked using 2 hours of incubation with a blocking solution [5% normal goat serum (NGS) and 1% Triton X-100 in PBS]. After blocking, sections were incubated at 4°C for 16 hours with a rabbit-anti-Piezo2 antibody (NBP1-78624, Novus Biologicals) at 1:500 dilution in 1% NGS and PBST. Postincubation in the primary antibody, the sections underwent three PBST washes for 15 min each. The sections were then incubated with a secondary antibody [Anti-Rabbit IgG (immunoglobulin G) Alexa 488, 18772-1ML-F, 1:1000 in 1% NGS and PBST] for 4 hours at room temperature. Postincubation with the secondary antibody, three PBST washes of 15 min each were given, and then the sections were stained with 4′,6-diamidino-2-phenylindole (DAPI; 1 μM in PBS). Stained sections were mounted on glass slides using VECTASHIELD (Vector Labs, H-1000) mounting media. The sections were imaged using a Leica Sp8 confocal microscope.

#### GAD65 staining

We used GAD65-EYFP animals to visualize the distribution of GAD65 (GAD2) interneurons in the mouse brain. Mice were perfused with 1× PBS and 4% PFA, and the dissected brain was stored at 4°C for 24 hours in 4% PFA. The brains were then cryopreserved in a 30% sucrose solution for 24 hours. Coronal sections (50 μm thick) were taken using a cryotome and collected in a 1× PBS–containing 24-well plate. Following sectioning, the sections received a 10-min 1× PBS wash. After incubating with DAPI (1:500) for 10 min, the free-floating sections were washed with 1× PBS for an additional 10 min. The sections were placed on glass slides and mounted with VECTASHIELD mounting media. The sections were imaged using a Leica Sp8 confocal microscope.

#### c-Fos staining of OB sections

A group of GAD65-EYFP mice (*n* = 6) was trained to distinguish between 0.6 LPM versus 0.3 LPM, and another group of GAD65-EYFP animals (*n* = 6) underwent a NORT. Three mice from each group were randomly processed for immunohistochemical analyses of c-Fos once they reached the asymptotic phase of learning. Mice were perfused with 1× PBS and 4% PFA, and the dissected brain was stored at 4°C for 24 hours in 4% PFA. Following fixation, brain tissue was submerged in 30% sucrose and embedded in Cryomatrix, and 50-μm sections were taken using a Leica Cryostate (CM1860B). These sections were washed three times for 15 min each with 1× tris-buffered saline (TBS). Blocking was performed for 2 hours to remove any nonspecific binding. The blocking solution contained 0.2% Triton X-100 (Sigma-Aldrich), 7.5% NGS (Abcam, ab7481), and 2.5% bovine serum albumin (Sigma-Aldrich) in TBS. Following blocking, a primary antibody was used: rabbit anti–c-Fos (1:750 dilution). The sections were incubated overnight at 4°C for 13 to 15 hours. The following day, three TBS washes (15 min each) were given. The secondary antibody was then incubated at room temperature for 2 hours. The secondary antibody used was anti-rabbit Alexa Flour 594 (1:1000 dilution) (Jackson Immunoresearch, US; code: 111-585-003). Three TBS washes (15 min each) were given. Last, the sections were stained with DAPI (Sigma-Aldrich, 1:750 dilution in 1% NGS) for 10 min and were mounted on the slides. The sections were imaged using a Leica Sp8 confocal microscope.

#### c-Fos + ve cell counting

Hygens professional software was used for cell counting using a custom workflow. In images, a three-dimensional ROI was defined, and all objects within the ROI were quantified. Any objects below 350 to 380 voxels were discarded as noise. Similar settings were uniformly applied to all images, with only minor adjustments to the threshold based on the relative intensity of the signal. The threshold was determined by focusing on cells in the external plexiform layer, where the c-Fos signal intensity was relatively lower compared to the GCL. After automated counting, clumps of cells with high voxel counts (e.g., more than 2600 voxels) were manually examined across different *z* planes to accurately assess the number of c-Fos–expressing cells. Cell counting was done for three animals with six sections (for each animal) and two or three ROIs per section.

#### Western blotting

We used 8- to 10-week-old GluA2-Lox (*n* = 5) and GAD65-GluA2 knockout (*n* = 6) animals for Western blotting. After being dissected, the OBs were stored at −80°C until they were needed for Western blotting. Whole-brain lysates were prepared in radioimmunoprecipitation assay buffer supplemented with a complete protease inhibitor. A bicinchoninic acid protein assay kit was used to perform the protein estimation. The sample (20 μg) was loaded in each well of a 10% polyacrylamide gel, and SDS–polyacrylamide gel electrophoresis was performed. The protein was then transferred to Immobilon-P polyvinylidene difluoride membranes. Blocking was then performed with 5% milk/TBS-Tween 20 for 1 hour at room temperature. The primary anti-GluA2 antibody and anti–glyceraldehyde-3-phosphate dehydrogenase (GAPDH) were used to probe the membranes for 16 hours at 4°C at 1:250 and 1:5000 dilutions, respectively. Peroxidase-conjugated AffiniPure Goat anti-rabbit IgG was used as the secondary antibody. It was used at 1:5000 dilution and incubated for 1 hour at room temperature. Using a Clarity ECL Western Blotting Substrate, a bound antibody was detected, and an ImageQuant LAS 4000 was used to digitally capture the image.

#### Microendoscopic Ca^2+^ imaging

Three groups of GAD65-GCaMP6f mice were used to perform Ca^2+^ imaging. The first group consisted of five animals that underwent calcium imaging while anesthetized, while the second group consisted of five to seven animals that underwent Ca^2+^ imaging while awake. The third group of four animals underwent complex odor discrimination.

#### Image acquisition

Calcium imaging was performed on animals while they were anesthetized and awake under head-restrained conditions. GAD65 + ve interneurons in the OB GCL were imaged using a snap-in fluorescence microscope (OSFM model L, Doric lenses Inc., Canada) mounted on an implanted GRIN cannula (1 mm in length). The cannula had a working distance of 80 μm and a focal range of 50 μm. At a frame rate of 10 Hz, a field of view corresponding to 350 μm by 350 μm that was further binned to 2 by 2 times was imaged. The CE:YAG fluorescence source of 465-nm output was used with power ranging from 400 to 700 mA.

For imaging under anesthetized conditions, an intraperitoneal injection of a ketamine-xylazine cocktail was administered to animals before the beginning of each session. Before mounting the animals on the setup, their toe reflexes were tested. Each session consisted of 60 to 80 trials with an ITI of 13.2 s. The animals (first group, *n* = 5 mice) were given different airflows with the stimuli of 100, 200, 300, and 400 ml/min, which were presented to them in a pseudorandomized fashion. Twenty trials per animal were conducted and analyzed for each airflow.

Animals were subjected to standard airflow-based discrimination tasks under head-restrained conditions for imaging under an awake state. Animals (second group, *n* = 5 to 7) underwent a task habituation phase and were trained to discriminate 0.5 LPM versus 0.4 LPM (four tasks, 1200 trials). Animals (third group, *n* = 4) also underwent a complex odor discrimination task where they were trained to discriminate a 60:40 mixture of octanol (+/−). Calcium traces were recorded during the first 40 trials (20 S+ and 20 S−) of each task (300 trials) at the beginning of each task. An external tone transistor-transistor logic signal (200 ms) from the olfactometer synchronized Ca^2+^ imaging to start of the trial. The imaging for each trial lasted 10 s, which included the stimulus duration of 2 s. The imaging was done at a resolution of 100 ms. The trial-by-trial imaging data were compiled by comparing the trial sequence generated by the olfactometer result file.

#### Imaging analysis

Custom-written Python scripts were used to analyze the images. The population activity was analyzed by selecting the ROIs of 300 μm by 300 μm. If there were any frame drops during image acquisition, such trials were identified and excluded from the analysis. The relative changes in fluorescence for each frame were calculated using *F*(*t*)/*F*_0_ = [*F*(*t*) − *F*_0_]/*F*_0_, where *F*_0_ is the mean activity during the baseline. The *F*_0_ was calculated trial-wise. The *F*(*t*)/*F*_0_ amplitude and AUC of individual animals were measured trial-wise.

### Optogenetics experiments

#### Optogenetic modulation of OB-specific GAD65-expressing interneurons

To investigate the effects of inhibition on M/T cells and their effects on airflow detection and discrimination behavior, we ontogenetically modulated the activity of OB-specific GAD65-expressing interneurons in a bidirectional manner by expressing ChR2 and Arch under the GAD65 promoter. Three different sets of animals were used for optogenetic experiments: first group, GAD65-Arch (*n* = 12 to 14); second group, GAD65-ChR2 (*n* = 7 to 10); third group, GAD65-EYFP (*n* = 12 to 14). While mice were performing airflow/odor discrimination, GAD65 + ve interneurons were photostimulated/photoinhibited. To activate the interneurons, we used 5-ms pulses of blue light (473 nm specific for ChR2) at a frequency of 40 Hz. To inhibit the interneurons, we used 1000-ms pulses of amber light (595 nm specific for Arch) at a 1-Hz frequency. For these experiments, the light stimulations were timed to coincide with the onset of the stimulus.

#### Task habituation phase and LED power standardization

After 1 week of LED implantation, the water deprivation schedule of animals began. Animals were subjected to task habituation training 3 or 4 days after the start of the water deprivation schedule. The operant conditioning paradigm used for these animals was the same as for the head-restrained task habituation phase. As optogenetic experiments were carried out to determine whether modulation of GAD65 + ve interneurons affects airflow discrimination behavior, and because the extent of transparency of the cranial window may vary between mice, the optimal power required to modulate the majority of GAD65 + ve interneuron activity would also vary. As a result, when the animals completed the task habituation, the strength of the LEDs was standardized using the detection task by varying the power of the LEDs during the task. The LEDs were powered by an LED driver, and the stimulus used to standardize the LED power was a 0.2 LPM airflow stimulus. While standardizing the LED intensity, we also ensured that the tissue temperature did not go beyond the body temperature (37°C). Using a laser power and energy meter, we measured the surface temperature of the LED and the wattage for each light power setting.

Animals were given the stimulus along with photodiode (LED) activation. The photoactivation-ON trials were interleaved with no-photoactivation trials (20 light-ON trials followed by 10 light OFF trials). Different light intensities were used, and 20 to 40 trials were performed for each LED intensity. Lick responses of animals were recorded and compared during stimulus presentation under light ON and OFF conditions. The average lick responses of the animals in GAD65-Arch and GAD65-ChR2 groups decreased as the intensity of LED increased (Fig. 5E). For subsequent optogenetic experiments, the LED intensities of the animals were chosen so that there was no difference between the light ON and OFF states, whereas the lick responses of the control EYFP animals did not alter (Arch, 2.93 mW; ChR2, 6.96 mW). Because GAD65-EFYP was used as a control group and expressed EYFP, which is also expressed in GAD65-ChR2, the intensity of light used for optogenetics training for these animals was the same as for the ChR2 group (6.96 mW). At this light intensity, there was no difference in the lick responses of these animals when compared to those under light OFF conditions (Fig. 5E).

#### Discrimination training

Animals were trained for airflow/odor discriminations under head-restrained conditions, and the paradigm and reward criteria for this task were the same as those mentioned in the section on behavioral training under head-restrained conditions. The whiskers of animals were kept trimmed throughout the experiment to rule out any possibility of whiskers being involved in airflow discrimination behavior. The whiskers were trimmed once a week. The animals were trained for different airflow and odor pairs. The sequence of training is summarized in Table 1.

**Table 1.** Training sequence of animals for various tasks under different photoactivation conditions.

Airflow pair/odor pair	Photoactivation
0.5 vs 0.4 LPM (1200 trials)	OFF
0.15 vs 0.1 LPM (900 trials)	ON
0.3 vs 0.25 LPM (600 trials)	OFF
0.45 vs 0.35 LPM (600 trials)	ON
0.6 vs 0.6 LPM (300 trials)	OFF
Octanol (+) (60%)-(−) (40%) versus octanol (−) (60%)-(+) (40%) (600 trials): 0.6 LPM for dilution of both stimuli	ON
Hexanal (60%)-pentanone (40%) (0.4 LPM) vs pentanone (60%)-hexanal (40%) (0.3 LPM) (600 trials)	ON
Carvone (+) (0.6 vs 0.4%) (600 trials): 0.2 LPM for dilution of both stimuli	ON

### Measurement of airflow in nature

The airflow was measured from the potential rodent habitats that include burrows, grocery shops, waste disposal sites, etc., with the help of a hot-wire anemometer acquired from PCE Instruments. The anemometer was kept inside the burrow, or the location of the potential habitat, and the airflow rate was averaged for a total of 100 s. The measurements were done from multiple locations and were pooled together to compute the airflow range that rodents encounter in nature.

### PID measurements

The PID measurements were done using an Aurora Scientific mini PID. The detection limit is 100 parts per billion of propylene in air, and the full-scale measurement range is 500 parts per million. PID measurements were taken to rule out the presence of any consistent odorant cues within the airflow stimulus. High-intensity photons colliding with vaporized molecules of odors, if present, would cause a voltage shift in the photoionic detector during PID measurements. PID measurements were performed for various airflows using a PID probe under similar conditions that were used for airflow detection and discrimination experiments. PID measurements were also performed on a set of odorants to demonstrate the difference that exists between airflow- and odor-generated PID patterns.

### Data and statistical analysis

GraphPad Prism 9, Microsoft Excel, and Python were used for all statistical analyses in this study. The data are presented as the means ± SEM. To determine *P* values and test for statistical significance, we used Student’s *t* test, one-way and two-way ANOVAs, and associated post hoc tests.
