# Supplementary material for: Mouse olfactory system acts as anemo-detector and anemo-discriminator
Source: Sci Adv. 2025 Oct 8;11(41):eadq8390. doi: 10.1126/sciadv.adq8390 (PMC12506969; doi:10.1126/sciadv.adq8390)
Supplement: Supplementary file 1 — Figs. S1 to S12 Legends for movies S1 and S2 [file sciadv.adq8390_sm.pdf]

Science Advances
AAAS

Supplementary Materials for

Mouse olfactory system acts as anemo-detector and anemo-discriminator

Sarang Mahajan *et al.*

Corresponding author: Nixon M. Abraham, nabraham@iiserpune.ac.in

Sci. Adv. **11**, eadq8390 (2025)
DOI: 10.1126/sciadv.adq8390

The PDF file includes:

Figs. S1 to S12
Legends for movies S1 and S2

Other Supplementary Material for this manuscript includes the following:

Movies S1 and S2

Supplementary Figures

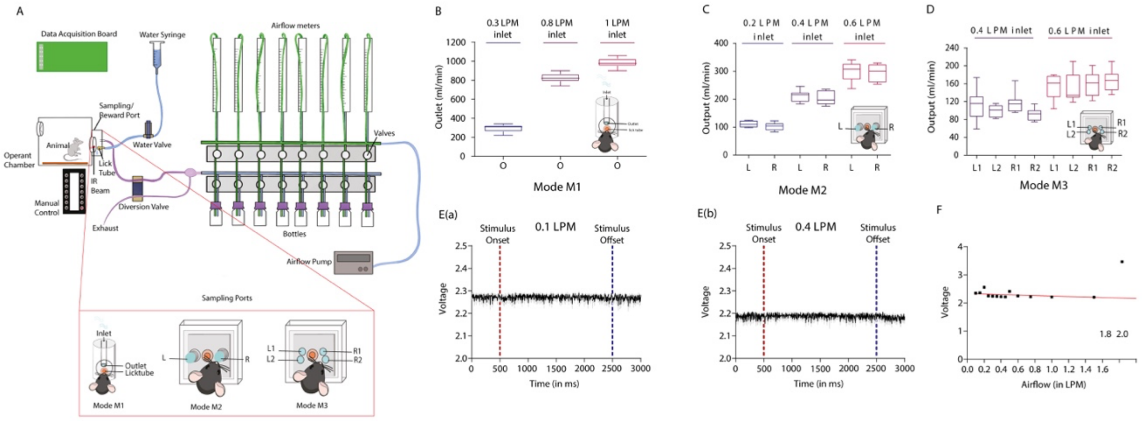

Figure S1. Flowrate and PID measurements for optimizing the airflow stimuli used for discrimination assays

(A) Illustration showing different components of a custom-built behavioral apparatus used to study anemo-detection and discrimination under freely-moving conditions. (**B)** Outlet airflow was measured from the stimulus delivery tube in the Mode M1 while different airflows were provided as the input. For 0.3 LPM (or 300 ml/min) inlet, the measured output was 296 ± 12.58 ml/min (Mean ± SEM). For 0.8 LPM inlet (or 800 ml/min), output was 793 ± 10.44 ml/min, and for 1.0 LPM (or 1000 ml/min) the output was 980 ± 15.78 ml/min. The input airflow rate supplied and the actual output rate observed were similar**. (C)** Outlet airflow was measured from different ports of the stimulus delivery tube in the Mode M2 while different airflows were provided as the input. For 0.2 LPM (or 200 ml/min) total inlet, the measured output was: Left port – 111.5 ± 3.625 ml/min and Right port – 102.8 ± 4.735, for 0.4 LPM (or 400 ml/min) total inlet, output was: Left port – 212.6 ± 7.131 ml/min and Right port – 203.5 ± 8.360, and for 0.6 LPM (or 600 ml/min) the output was: Left port – 303.3 ± 12.09 ml/min and Right port – 296.5 ± 10.62. The airflow rates through left and right port for a particular inlet stimulus were not different and their sum were similar to that of the inlet airflow. **(D)** Outlet airflow was measured from different ports of the stimulus delivery tube in the Mode M3 while different airflows were provided as the input. For 0.4 LPM (or 400 ml/min) total inlet, the measured output was: Left port1 – 112.6 ± 12.23 ml/min, Left port 2 – 99.25 ± 4.758, Right port 1 – 117.5 ± 8.082 and Right port2 – 92.50 ± 4.679, for 0.6 LPM (or 600 ml/min) total inlet, output was: Left port1 – 154.5 ± 9.716 ml/min, Left port 2 – 151.3 ± 11.40, Right port 1 – 159.6 ± 9.365 and Right port2 – 166.6 ± 8.294. The airflow rates through all the ports for a particular inlet stimulus were not different and their sum were similar to that of the inlet airflow. **E(a,b)** PID profiles of 0.1 LPM and 0.4 LPM. For both airflows, the voltage in the PID profiles remains unchanged after the stimulus onset. **(F)** Curve representing change in amplitude voltage of different airflows with respect to their stimulus strength. The amplitude and stimulus strength exhibited no correlation (Pearson R^2 = 0.2176) as well as no linear trend (Linear Regression R^2 = 0.1572).

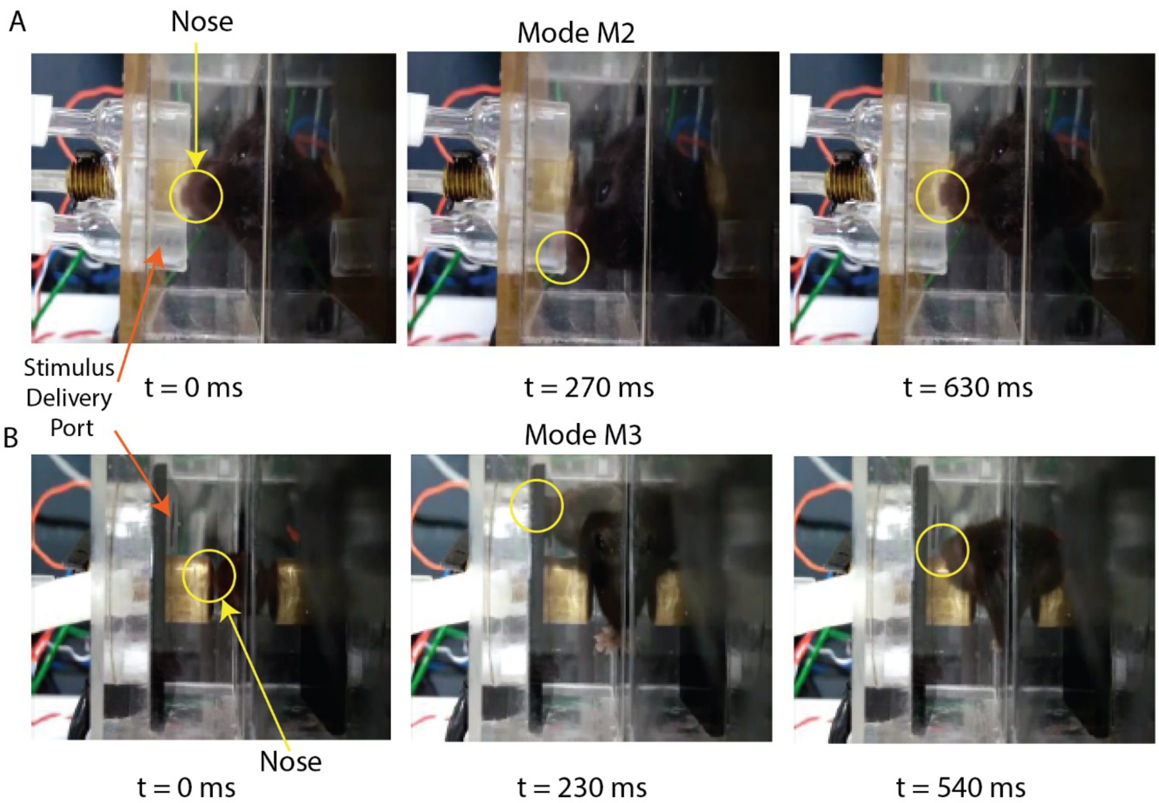

Figure S2. Animals use their nose to sample the stimuli while performing airflow discrimination task.

(A) The top row depicts snapshots taken at different time points during a trial for M2 mode. The mouse inserts its nostril into the stimulus port at t= 270 ms, suggesting using the nose to sample the stimulus. **(B)** Bottom row depicts snapshots taken at different time points during a trial for M3 mode. In this mode, the mouse inserts its nostril into the stimulus port at t= 230 ms.

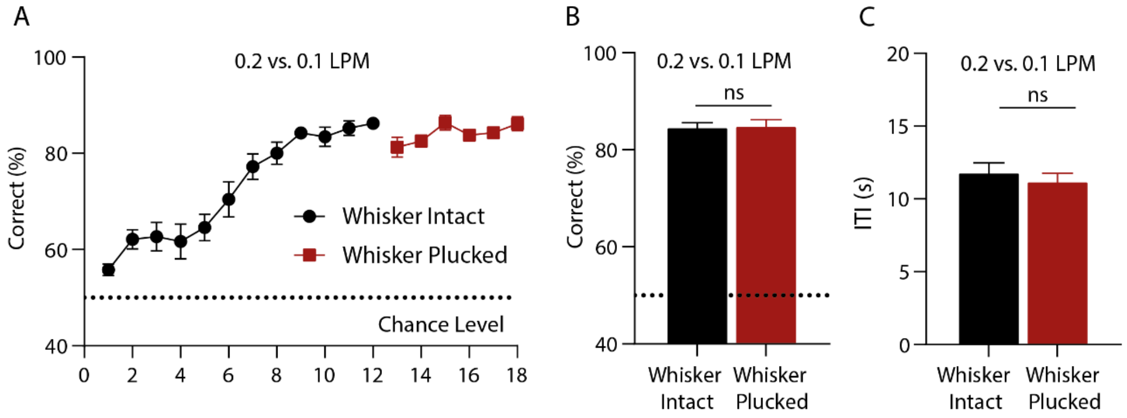

Figure S3. Performance of animals on 0.2 vs. 0.1 LPM before and after whisker plucking.

(A) Learning curve before and after whisker plucking. Animals reached the asymptotic phase of learning under both conditions. **(B)** Final accuracies of animals (average of last 300 trials) before and after whisker plucking. The accuracies were found to be similar (Before: 84.96 ± 1.261; After: 84.71 ± 0.8578, two-tailed paired t-test, p = 0.7550, n = 9). **(C)** Final ITIs of animals (average of last 300 trials) before and after whisker plucking. The ITIs were found to be similar (Before: 11.22 ± 0.5317 s, After: 11.84 ± 0.6322, two-tailed paired t-test, p = 0.3043, n = 9).

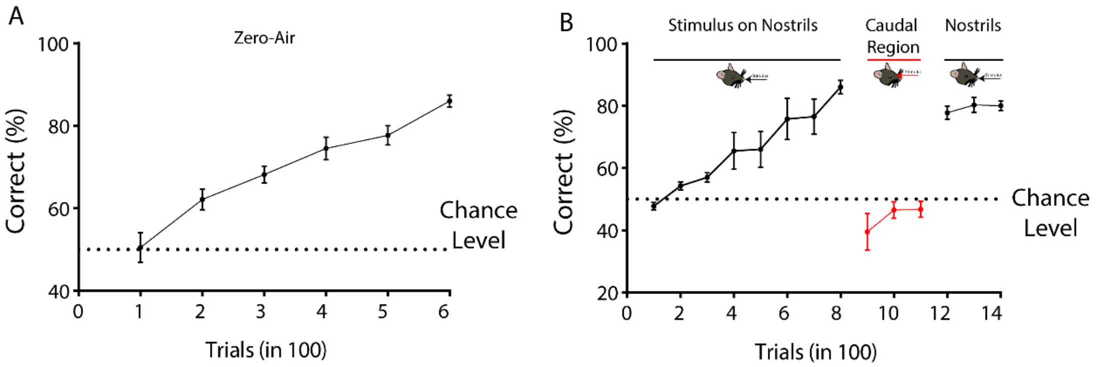

Figure S4. Performance of animals on different airflow discrimination tasks.
(A) Learning curve of animals when trained to discriminate 1.5 vs. 0.75 LPM delivered through an air-cylinder. Animals showed final accuracy of >80% (n = 6) **(B)** Animals were trained to discriminate 0.6 vs. 0.3 LPM under head-restrained conditions. Once animals showed an accuracy of >80%, the airflow was diverted from the nostrils to the caudal region. Animals showed performance deficits under these conditions (Red learning curve). The learning was restored while the stimulus was redirected to the nostrils (n = 4).

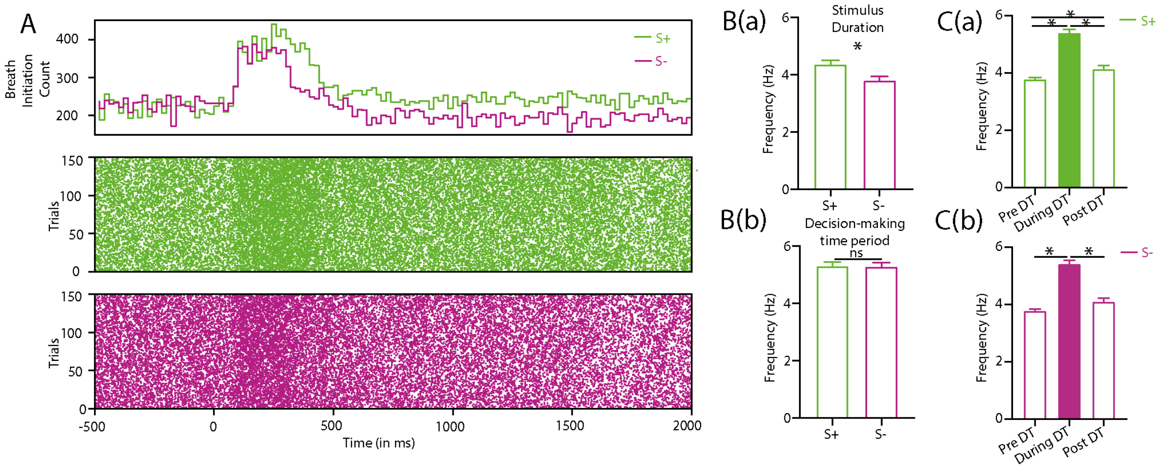

Figure S5. Sampling behavior of animals for S+ and S- trials pooled across airflow pairs
(A) Raster plots and histograms representing inhalation onset for S+ and S- trials pooled across all airflow pairs. **B(a)** Bar graphs representing sniff frequencies of animals for S+ and S- trials during 2s stimulus duration. The SFs of animals for S+ trials were significantly higher than S- trials (two-tailed paired t-test, p < 0.0001, n = 5-8 mice). **B(b)** Bar graphs representing sniff frequencies of animals for S+ and S- trials during the decision-making window. The SFs of animals for S+ and S- trials were similar (two-tailed paired t-test, p = 0.8663, n = 5-8 mice). **C(a)** Bar graphs representing sniff frequencies of animals for S+ trials for pre-, during and post-decision-making period. The SFs of animals during the decision-making period were higher (one-way repeated measures ANOVA with Tukey's multiple comparison test, F = 84.92, p < 0.0001, n = 5-8). **C(b)** Bar graphs representing sniff frequencies of animals for S- trials for pre-, during and post-decision-making period. The SFs of animals during the decision-making period were higher (one-

way repeated measures ANOVA with Tukey's multiple comparison test, $F = 68.19$, $p < 0.0001$, $n = 5\text{-}8$).

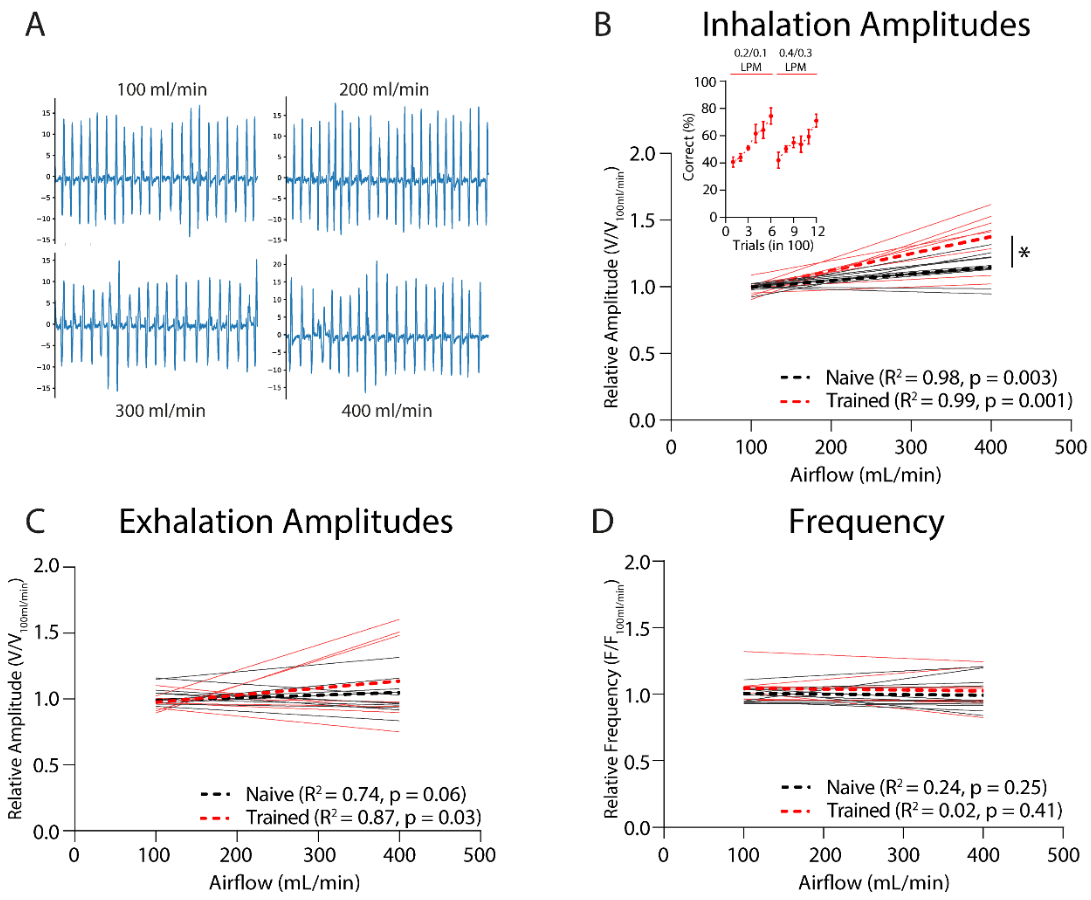

Figure S6. Stimulus-dependent variation of sniffing was recorded within the nasal cavity of naïve and learned mice.

(A) Representative traces for each flow for a single trial. (B) Graph demonstrating an increase in relative amplitudes for inhalation along with stimulus strength (Black continuous lines represent traces for individual animals, and the black dotted line is the correlation for all the animals pooled). We observed a significant correlation for 8/11 naïve and 6/8 trained mice in individual mouse analysis. For black dotted line: Pearson Correlation, $R^2 = 0.98$, $p = 0.003$, and for red dotted line: Pearson Correlation, $R^2 = 0.99$, $p = 0.001$). The inset graph represents the airflow discrimination learning. This Panel (B) is also part of the main figure (Fig. 3J) (C) Graph demonstrating changes in relative amplitudes for exhalation along with stimulus strength. We did not observe changes for most of the naïve and trained mice in individual mouse analysis., (For black dotted line: Pearson Correlation, $R^2 = 0.74$, $p = 0.06$, For red dotted line: Pearson Correlation, $R^2 = 0.87$, $p = 0.03$). (D) Graph demonstrating no change in relative frequency along with stimulus strength (For black line: Pearson Correlation, $R^2 = 0.24$, $p = 0.25$, For red dotted line: $R^2 = 0.02$, $p = 0.41$).

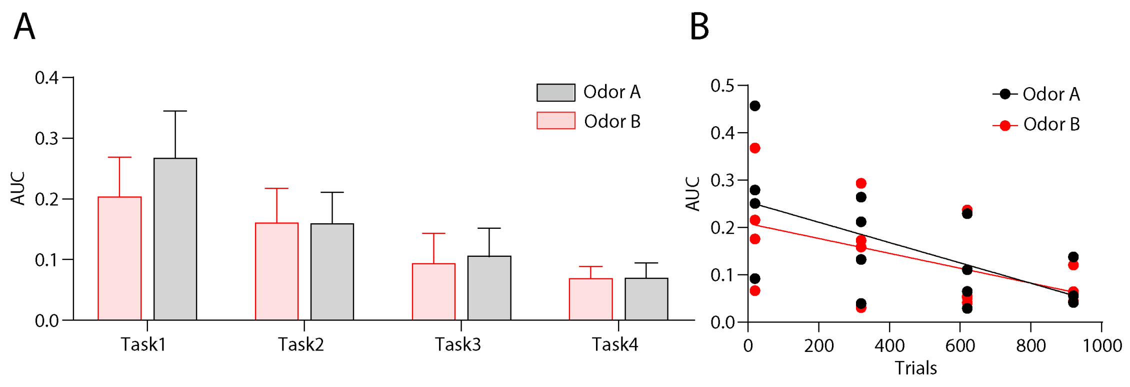

Figure S7. Calcium dynamics in GAD65-expressing interneurons during a complex odor discrimination task.

(A) Bar graphs representing average AUC of animals for Odor A and Odor B stimuli with progression of trials. As learning progressed, a decrease in AUCs was observed. **(B)** Plot representing change in average AUC of animals for Odor A and Odor B stimuli with number of trials. For both stimuli, change in average AUC showed a negative correlation with learning (Odor A: R2 = 0.93, p = 0.03; Odor B: R2 = 0.97, p = 0.01, n = 4). Odor A – Hexanal (60%) + Pentanone (40%), Odor B - Hexanal (40%) + Pentanone (60%).

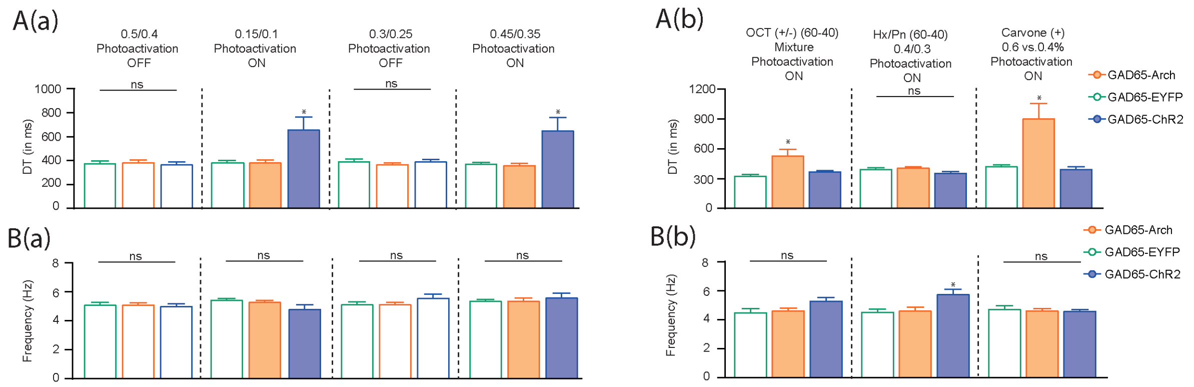

Figure S8. Discrimination time and sniff frequency of animals for airflow and odor-based tasks under different photoactivation conditions.

A(a) Bar graphs representing average DTs for all groups of animals for various airflows under different photoactivation conditions. The DTs of animals under photoactivation conditions for ChR2 group was slower compared to other groups of animals. (One-way ANOVA with Tukey's multiple comparison test * represents p < 0.05). **A(b)** Bar graphs representing average DTs for all groups of animals for various odor-based tasks. One-way ANOVA with Tukey's multiple comparisons. (* represents p < 0.05). **B(a)** Bar graphs representing average sniff frequencies for all groups of animals during discrimination time for various airflows under different photoactivation conditions. No differences in the SF for all the groups under different photoactivation conditions were observed. (One-way ANOVA with Tukey's multiple comparison test * represents p < 0.05). **B(b)** Bar graphs representing average sniff frequencies during discrimination time for all groups of animals for various odor-based tasks. One-way ANOVA with Tukey's multiple comparisons. (* represents p < 0.05).

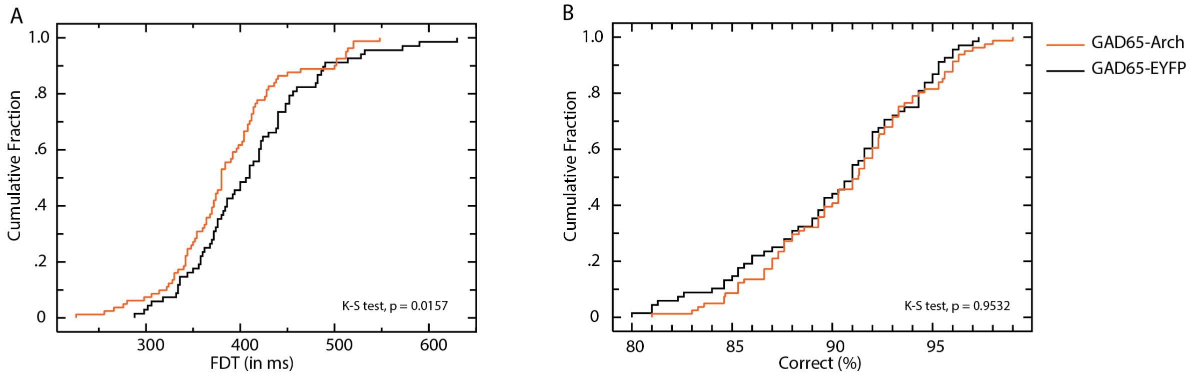

Figure S9. GAD65-Arch animals showed faster discrimination times under photoactivation conditions for airflow discrimination task.
(A) Cumulative fraction representing the frequency corresponding to a specific DT for different groups. Orange line represents cumulative fraction of DTs for GAD65-Arch group, whereas black line represents the same for GAD65-EYFP group. Arch animals made faster decisions under photoactivation conditions (K-S, D-value = 0.256, p = 0.0157). **(B)** Cumulative fraction representing the frequency corresponding to a specific accuracy for different groups. Orange line represents cumulative fraction of accuracies for GAD65-Arch group, whereas black line represents the same for GAD65-EYFP group. The accuracies of both groups of animals were similar (K-S, D-value = 0.0848, p = 0.9532).

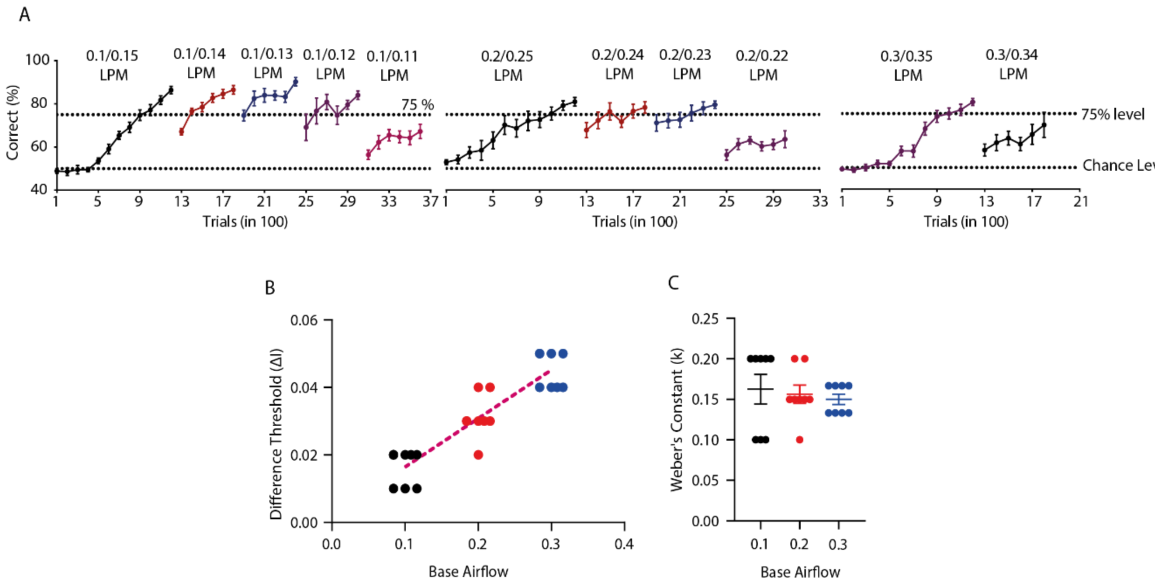

Figure S10. Airflow discrimination via olfactory system follows Weber's Law.
(A) Finding difference threshold during airflow discrimination. Different groups of animals were trained to discriminate various airflow rates (0.1 vs. 0.15 LPM, 0.1 vs 0.14 LPM, 0.1 vs. 0.13 LPM, 0.1 vs. 0.12 LPM, 0.1 vs. 0.11 LPM) in order to calculate the discrimination threshold or just noticeable difference (JND_{75}) for 0.1 LPM, while the difference threshold was kept at 75% accuracy. Similar is done with other two groups of animals trained to find the JND for 0.2 and 0.3 LPM as well. **(B)** Difference threshold (ΔI) for different base airflows. Points represent values of

ΔI for individual animals. ΔI positively correlates with increase in the airflow base value (R^2 = 0.9944, p = 0.060). Magenta dotted line shows linear regression with R^2 = 0.8300 and slope = 0.1438) (n = 8 for all three base airflows). **(C)** Weber's constant (k) calculated for different base airflows. The dots represent individual values whereas horizontal line represents mean along with SEM (one-way ANOVA, F = 0.2330, p = 0.7941) (n = 8 for all three base airflows).

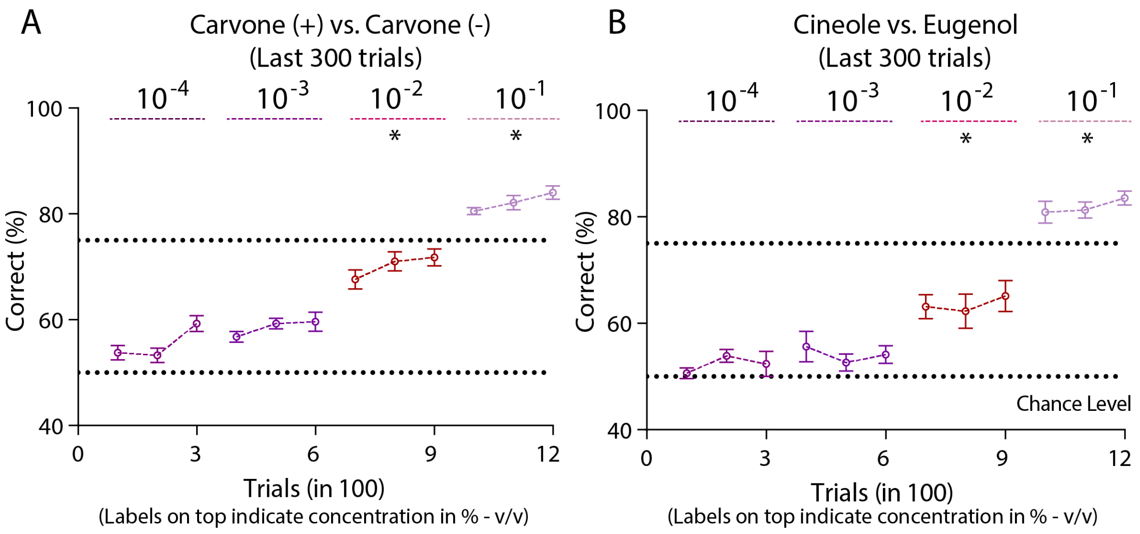

Figure S11. Discrimination threshold of animals during odor-based discrimination task.
(A) Animals were trained to discriminate different concentrations of Carvones (+) vs. Carvones (-) to quantify the discrimination threshold. Animals were trained on 10^{-4} % (v/v), 10^{-3} %, 10^{-2} %, and 10^{-1} % concentrations. The graphs represent the learning curve for last 300 trials when trained on a particular concentration. The performance of animals was subthreshold (<75%) for all the concentrations except to that of 10^{-1} %. **(B)** Animals were trained to discriminate different concentrations of Cineole vs. Eugenol to quantify the discrimination threshold. Animals were trained on 10^{-4} % (v/v), 10^{-3} %, 10^{-2} %, and 10^{-1} % concentrations. The graphs represent the learning curve for last 300 trials when trained on a particular concentration. The performance of animals was subthreshold (<75%) for all the concentrations except to that of 10^{-1} %.

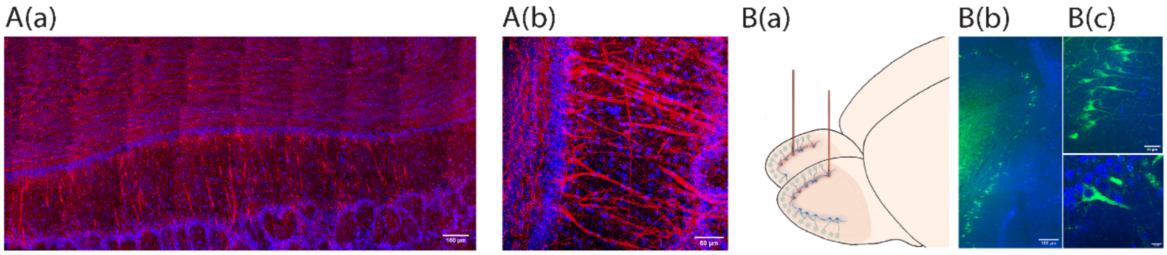

Figure S12. Peizo-2 expression in the olfactory bulb.
A(a) Peizo-2 expression in a coronal section of the olfactory bulb. **A(b)** Peizo-2 expression in the mitral cell layer. **B(a)** Schematic representing injection of FM 1-43 dye. **B(b,c)** Images representing the uptake of FM 1-43 dye in the OB mitral cells.

Supplementary Movies

Movie S1. Animal performing an airflow discrimination task with the stimulus delivered via Mode M2.

Movie S2. Animal performing an airflow discrimination task with the stimulus delivered via Mode M3.

For both M2 and M3 modes, the trial begins when the animal pokes its head into the sampling port, triggering the delivery of airflow through the stimulus delivery ports. The animal can be seen using its snout to sample the airflow and decide whether to lick for a reward or retract its head.
